# A Recyclable Inert Inorganic Framework Assisted Solid-State Electrolyte for Long-Life Aluminum Ion Batteries

**DOI:** 10.1021/acscentsci.4c01615

**Published:** 2024-12-19

**Authors:** Ke Guo, Wei Wang, Wei-Li Song, Shijie Li, Xueyan Du, Shuqiang Jiao

**Affiliations:** † Institute of Advanced Structural Technology, Beijing Institute of Technology, Beijing 100081, China; ‡ State Key Laboratory of Advanced Metallurgy, University of Science and Technology Beijing, Beijing 100083, China; § State Key Laboratory of Advanced Processing and Recycling of Nonferrous Metals, Lanzhou University of Technology, Lanzhou 730050, China

## Abstract

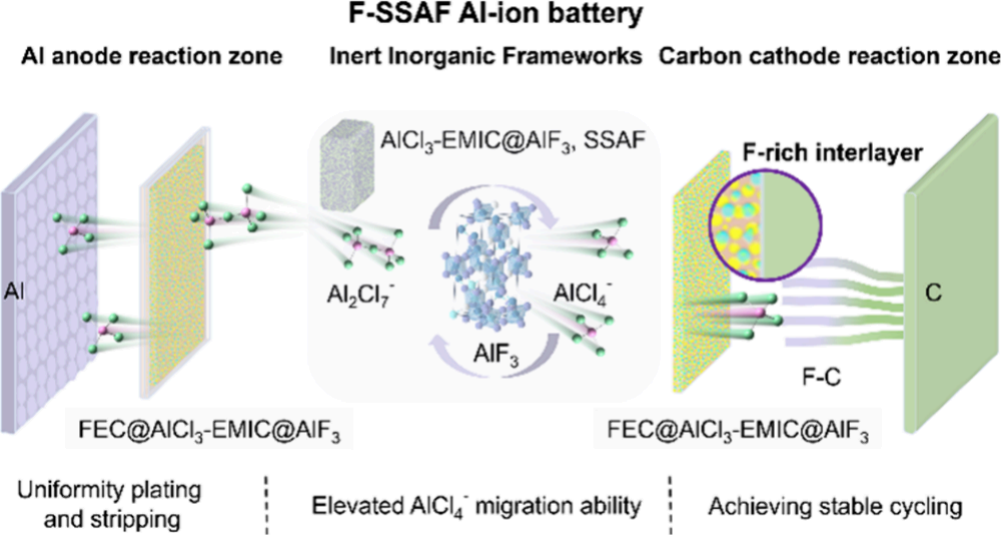

The environmentally friendly and high-safety aluminum-ion batteries (AIBs) have attracted intense interest, but the extensive use of expensive EMIC-AlCl_3_ electrolyte, strong moisture sensitivity, and severe corrosion of the Al anode limit their commercial application. Herein, we develop a solid-state electrolyte (F-SSAF) with an AlF_3_ inert inorganic framework as the solid diluent, EMIC-AlCl_3_ as the electrolyte, and FEC@EMIC-AlCl_3_ (FIL) as the interface additive for solid-state AIBs (SSAIBs). The dissociation of Al_2_Cl_7_^–^ (AlCl_3_–AlCl_4_^–^) into AlCl_4_^–^ is promoted by AlF_3_, which can facilitate the migration rate of AlCl_4_^–^ active ions and simultaneously mitigate the corrosion of the Al anode. The introduction of an AlF_3_ inert inorganic framework can also reduce the dosage of expensive EMIC-AlCl_3_ and alleviate the moisture sensitivity of EMIC-AlCl_3_. The FIL is introduced into the surfaces of both anode and cathode, thus in situ forming F-rich SEI and CEI films. The F-SSAF enables Al|F-SSAF|Al symmetric cells to achieve ultralong stable deposition and dissolution of Al up to 4000 h, and Al|F-SSAF|C full cells to achieve an unprecedented long cycle life of 10000 cycles with an average Coulombic efficiency of >99%. In addition, up to 80% of the AlF_3_ inert inorganic framework can be recycled. This work provides a simple yet substantial strategy for low-cost, long-life, and high-safety SSAIBs.

## Abstract 2

A solid-state electrolyte (F-SSAF) with a recyclable AlF_3_ inert inorganic framework, EMIC-AlCl_3_ as electrolyte, and FEC@EMIC-AlCl_3_ (FIL) as interface additive has been developed for Al-ion Battery.

## Introduction

Lithium-ion batteries (LIBs) are widely used in portable devices, electric vehicle and energy storage systems (ESSs) because of their high energy density and mature industrial preparation systems.^1−4^ However, the application of LIBs technology to large-scale ESSs poses challenges, such as limited resource availability, cost-ineffectiveness, and especially safety risk.^5,6^ Given the global push toward sustainability, exploring abundant and renewable resources for advancements in energy storage battery technologies holds strategic significance for fostering sustainable development. In recent years, various alkali metal ion batteries (AMIBs) such as sodium-ion and potassium-ion batteries, along with calcium-ion, magnesium-ion, and zinc-ion batteries, etc., have garnered widespread attention from both industry and academia.^7−9^

Among the state-of-the-art battery systems, rechargeable aluminum-ion batteries (AIBs) with nonflammable room-temperature chloroaluminate-based ionic liquid electrolytes possess potential advantages of exceptional safety, long cycle life, and wide temperature adaptability, which makes AIBs highly suitable for large-scale energy storage systems.^10−16^ However, conventional ionic liquid electrolytes (ILs) in AIBs are plagued by notable vulnerabilities such as pronounced susceptibility to water vapor and strong corrosivity that lead to electrolyte leakage. In addition, unlike electrolytes used in AMIBs, the strong Lewis acidity of ILs makes it difficult to establish stable solid–electrolyte interphases (SEI) and cathode–electrolyte interfaces (CEI). Thus, to tackle these issues, researchers have embarked on a series of investigations of solid-state electrolytes (SSEs) for AIBs. Generally, gel polymer electrolytes (GPEs) are predominantly utilized in AIBs due to their notable advantages such as excellent interfacial contact, minimized leakage risk, and enhanced mechanical stability.^17−19^ For example, Kim et al. reported a novel GPE (ethyl acrylates in EMIC-AlCl_3_) for AIBs, significantly enhancing the cycle stability with about 95% capacity retention (500 cycles) and exhibiting robust mechanical performance and high safety under stringent operational conditions.^20^ These intrinsic properties make GPEs suitable for AIBs, yet the limitation, such as low ion conductivity and poor rate performance, is also obvious. In comparison, metal–organic frameworks (MOFs)-based electrolytes manifest exceptional ILs absorption ability within AIBs, which is attributed to their structural adjustability, inherent high porosity and extensive surface area.^21^ However, it is vital to acknowledge that both GPEs and MOFs fundamentally fall under the category of organic frameworks, and they unfortunately bring about some inherent challenges, such as poor thermal stability, low ionic conductivity, and complicated fabrication process. In this case, if these organic frameworks are replaced by inorganic frameworks, it can not only maintain the electrochemical performance of the EMIC-AlCl_3_ but also improve the mechanical and thermal stability of the electrolytes.^22,23^ Till now, the concept of using inert inorganic frameworks as solid diluent has been hardly ever reported in SSAIBs.

To fill this gap, we design a solid-state electrolyte (F-SSAF) with EMIC-AlCl_3_ as the electrolyte and AlF_3_ inert inorganic framework as the solid diluent. The SSAF electrolyte exhibits high ionic conductivity (7.0 mS cm^–1^), high anion-ion transference number (0.50), relaxative Al anode corrosion, low manufacturing cost, and alleviated moisture sensitivity. The 1 vol% FEC(Fluoroethylene carbonate)@EMIC-AlCl_3_ (FIL) as interface additive is introduced into the surfaces of both the anode and cathode, and F-rich SEI and CEI films are in situ formed, which further uniform the Al deposition/dissolution and inhibit the growth of Al dendrite. The Al|F-SSAF|Al symmetric cells show ultralong stable deposition and dissolution of Al up to 4000 h, and the assembled SSAIBs achieve 10000 cycles. Furthermore, AlF_3_ can be recycled from the used F-SSAF electrolyte with yields of >80%, while the Al foil can be directly used again only after a simple surface cleaning process. Future directions for the development of AIBs will focus on improving energy density, cycle life, and electrolyte stability, while the development of advanced electrodes, scalable production, and cost-effective solutions will be key to practical applications.

## Results and Discussion

### Electrochemical Characterization of Electrolytes

**Figure 1:**
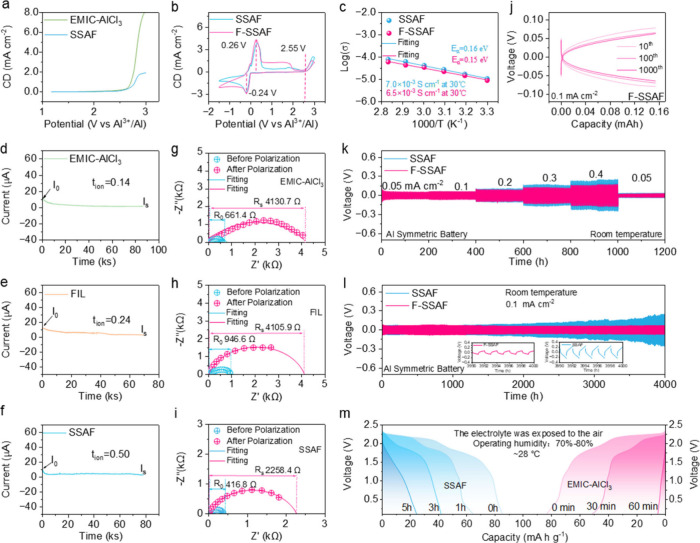
Electrochemical characterization of electrolytes. (a) LSV curves of the Al||Mo cells using EMIC-AlCl_3_ and SSAF electrolytes; CD: current density. (b) CV curves of the Al||Mo cells using SSAF and F-SSAF electrolytes. (c) Arrhenius conductivity plot of SSAF and F-SSAF electrolytes. Anion transference number tests of EMIC-AlCl_3_, FIL, and SSAF. Polarization (POL) result (d) and fitted EIS before and after the POL of EMIC-AlCl_3_ (g), POL result (e) and fitted EIS before and after the POL of FIL (h), and POL result (f) and fitted EIS before and after the POL of SSAF (i). (j) The voltage profiles (10th, 100th, 1000th cycles) of the Al|F-SSAF|Al symmetric cell. (k) Comparison of Al deposition/dissolution cycling processes for Al|SSAF|Al and Al|F-SSAF|Al symmetric cells at different current densities. (l) Galvanostatic Al deposition/dissolution curves of Al|SSAF|Al and Al|F-SSAF|Al symmetric cells at 0.1 mA cm^–2^, insets: the magnified curves during 3990–4000 h. (m) The discharge curves of Al||C cells using the SSAF and EMIC-AlCl_3_ electrolytes exposed to air for different time.

The SSAF electrolyte is prepared using EMIC-AlCl_3_ as electrolyte and the low-cost AlF_3_ inert inorganic framework as solid diluent. To evaluate the electrochemical stabilities of SSAF in SSAIBs, linear sweep voltammetry (LSV) tests are conducted. The oxidation potentials of SSAF are higher than those of EMIC-AlCl_3_, which demonstrates a larger potential window of SSEs (Figure 1a). Simultaneously, the oxidation potentials of F-SSAF are lower than those of SSAF, indicating that the FIL preferentially engages in interfacial reactions (Figure S1). Meanwhile, the cyclic voltammetry (CV) tests of the electrolytes are conducted by assembling SSAF and F-SSAF into Al||Mo cells, where Mo serves as a metal current collector (Figure 1b). Obvious oxidation and reduction peaks at 0.26 V and −0.24 V are observed, corresponding to the deposition and dissolution reactions of Al metal.^21^ Additionally, the decomposition potential of the two electrolytes is observed at 2.55 V, which agrees well with the LSV curves.

Ionic conductivity is a key parameter for the SSAIBs. To assess the ion conductivity of SSAF under different temperatures, electrochemical impedance spectroscopy (EIS) measurements are conducted using the configuration of Mo||Mo cells in a frequency range of 0.1–10^6^ Hz at 30–80 °C. The SSAF electrolyte exhibits an ionic conductivity of 7.0 mS cm^–1^ at 30 °C and 16.7 mS cm^–1^ at 80 °C, respectively (Figure 1c and Table S1). It is noteworthy that the activation energies of SSAF and F-SSAF electrolytes are 0.16 and 0.15 eV, respectively, indicating a fast AlCl_4_^–^ migration. Furthermore, the ionic conductivity and Arrhenius activation energy of SSAF with different EMIC-AlCl_3_ ratios (45 wt%, 50 wt%, 55 wt%) are further elucidated (Figure S2 and Table S2). With the increase in AlF_3_ in EMIC-AlCl_3_, a downward trend in ion conductivity and Arrhenius activation energy is observed.

The ion transference number (t_–_) is a pivotal parameter in assessing the rate capability of SSAIBs, as a diminished t_–_ indicates an elevated electrode polarization and decreased energy density. Figures 1d-f depicts the time-dependent response of the direct-current polarization for EMIC-AlCl_3_, FIL and SSAF, while Figures 1g-i shows the EIS curves before and after polarization. The calculated t_–_ of EMIC-AlCl_3_, FIL and SSAF is 0.14, 0.24 and 0.50, and the corresponding calculation details are provided in Table S3. It can also be deduced that the introduction of inert inorganic frameworks as solid diluent can greatly improve the ion transference number of active ions.

The long-term cycling stability of the electrolytes is further confirmed in Al||Al symmetrical cells. The voltage profiles for the 10th, 100th, and 1000th cycles are compared (Figures 1j and S3). Meanwhile, we also analyze and compare the rate capability of SSAF and F-SSAF electrolytes in Al||Al symmetrical cells (Figure 1k). The polarization voltage of the Al||Al symmetric cell in F-SSAF gradually decreases in the initial cycle at 0.05 mA cm^–2^ and the cell is cycled at step-increased current densities (0.1 mA cm^–2^ pre step) for a fixed 1.0 h to explore the Al dendrite suppression capability with FIL. The F-SSAF exhibits a smaller overpotential during each 200 h-cycling, which indicates an improved interface after the addition of FIL. The symmetrical cells are also tested at a room temperature under a current density of 0.1 mA cm^–2^, demonstrating long cycling stability for over 4000 h while maintaining a nearly constant polarization voltage (Figure 1l). To highlight the effect of the addition of FEC in EMIC-AlCl_3_ and the optimized proportion of 1 vol% FEC in EMIC-AlCl_3_, we carry out similar tests using EMIC-AlCl_3_ and FEC (1 vol%, 2 vol% and 3 vol%)@EMIC-AlCl_3_ electrolytes (Figure S4). After adding the FEC additive in EMIC-AlCl_3_, Fourier transform infrared (FT-IR) spectra show that C=O (1708 cm^–1^), C–O–C (1235 cm^–1^) and C–F (915 cm^–1^) peaks are enhanced with increased FEC concentration. And ^27^Al and ^19^F NMR spectra also show a new single Al–F signal, suggesting a polymerization reaction between FEC and EMIC-AlCl_3_ (Figure S5). This is basically consistent with the reported literatures.^24,25^ Therefore, the excessive incorporation of FEC (3 vol%) into the EMIC-AlCl_3_ electrolyte leads to a pronounced consumption of AlCl_3_. This reduces the concentration of anions, which, in turn, adversely affects the reversibility of aluminum deposition. The air stability of SSAF and EMIC-AlCl_3_ electrolytes is compared when they are exposed to high-humidity conditions (Figures 1m and S6). Notably, the capacity decays extremely when the cells with EMIC-AlCl_3_ are exposed to air for 1 h, while the capacity retention still maintains 75% when using SSAF as electrolyte.

### Electrochemical Characterization and Structure Evolution of Al||C Cells

**Figure 2:**
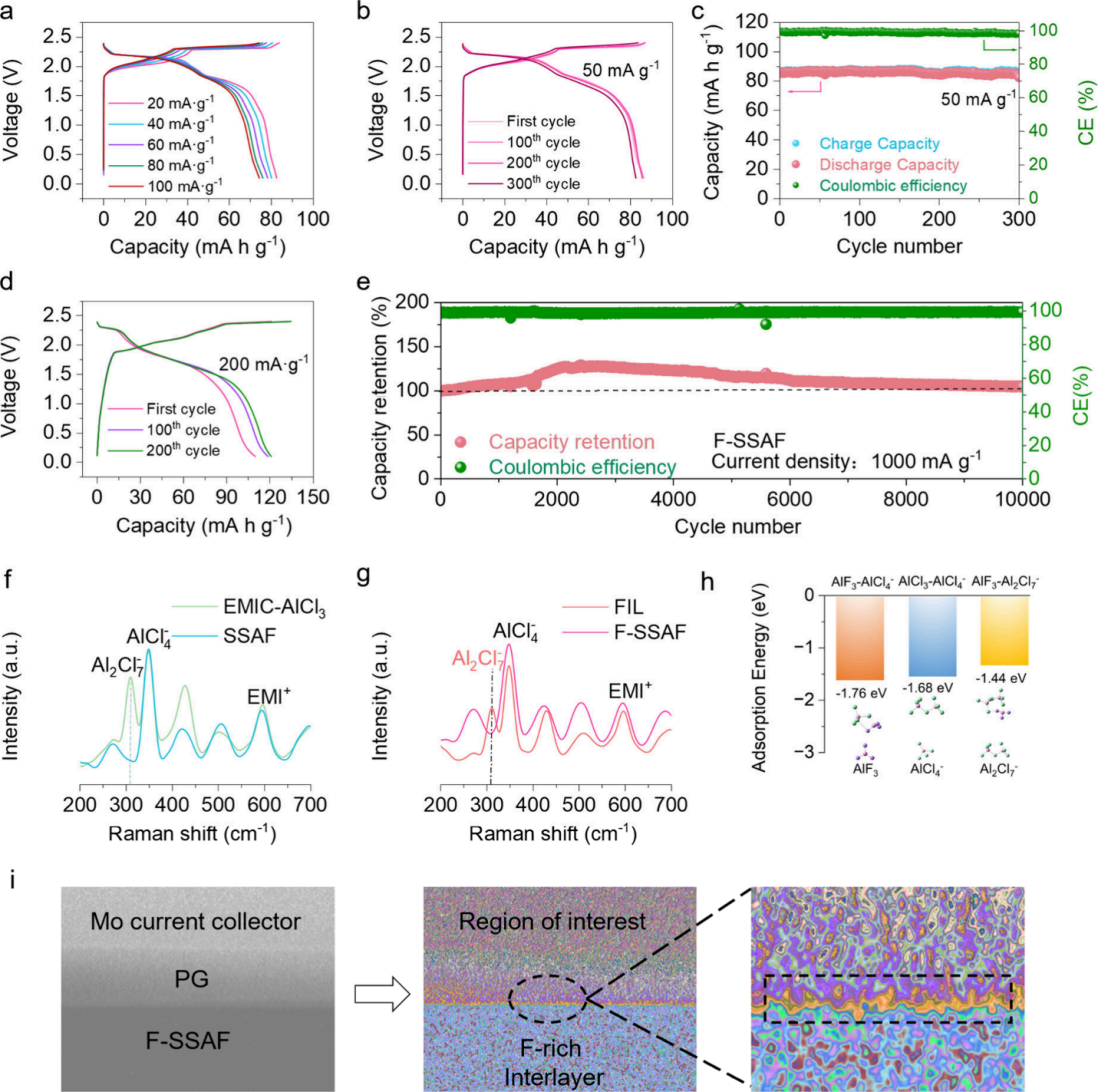
Electrochemical characterization and structure evolution of Al||C cells. The charge/discharge voltage profiles of Al|F-SSAF|PG cells under different current densities (a) and cycles (b). (c) The cycling stability of Al|F-SSAF|PG cells. The charge/discharge voltage profiles (d) and the discharge capacity retention (e) of Al|F-SSAF|graphite cells. (f-g) Raman spectra of different electrolytes. (h) Comparison of the adsorption energy of AlF_3_–AlCl_4_^–^, AlCl_3_–AlCl_4_^–^ and AlF_3_–Al_2_Cl_7_^–^. (i) The contrast enhancement analysis of the cathode-electrolyte interface using XR-CT.

To evaluate the cycling performance of the F-SSAF electrolyte, Al||C cells are assembled. And the electrolyte optimization details are studied in the assembled cells (Figures S7–S9). The cell with F-SSAF electrolyte and pyrolytic graphite (PG) cathode show reversible capacities of 83, 81, 79, 77, and 75 mA h g^–1^ at the current densities of 20, 40, 60, 80, and 100 mA g^–1^ (Figure 2a). The long cycling performances of the cells with different electrolytes are also compared (50 mA g^–1^, 0.1–2.4 V, room temperature) (Figures 2b, c and S8, S9). The cycling stability of Al||PG cells with the F-SSAF electrolyte shows a high capacity retention of 96.4% after 300 cycles. In addition to the favorable rate capability, the charge/discharge voltage profiles demonstrate a high reversibility^20^ (absence of hysteresis) even at a small current density of 10 mA g^–1^ (Figure S10). To analyze the electrochemical performance of the batteries, CV tests of EMIC-AlCl_3_, FIL, SSAF and F-SSAF at 0.2, 0.4, 0.6 0.8, 1.0 mV s^–1^ were carried out (Figure S11). This further confirms that the introduction of FEC additive into the SSAF electrolyte surface enhances the ion transference ability and the stability of the electrolyte-electrode interface. To verify the application universality of the designed electrolyte, a graphite cathode with F-SSAF electrolyte also exhibits a high capacity of 121 mA h g^–1^ at a current density of 200 mA g^–1^ (Figure 2d). To our surprise, an unprecedented long cycle life of 10000 cycles with high capacity retention and Coulombic efficiency is achieved (Figure 2e). Meanwhile, the Al||Graphite battery with the F-SSAF electrolyte also delivers an excellent rate performance, even at a high current density of 5 A g^–1^ (Figure S12).

To further understand the excellent properties of the electrolyte in cells, Raman spectroscopy and density functional theory (DFT) calculations are employed. The Raman spectra of EMIC-AlCl_3_ and FIL electrolytes emerge the peaks of AlCl_4_^–^ (349 cm^–1^), Al_2_Cl_7_^–^ (310 cm^–1^), and EMI^+^ (597 cm^–1^), but the peak of Al_2_Cl_7_^–^ disappears in both SSAF and F-SSAF electrolytes^26^ (Figures 2f, g). We speculate that the introduction of AlF_3_ could promote the dissociation of Al_2_Cl_7_^–^ (AlCl_3_–AlCl_4_^–^) into AlCl_4_^–^. To further prove our hypothesis, DFT calculations show that the adsorption energy between AlF_3_ and AlCl_4_^–^ is higher than that between AlCl_3_ and AlCl_4_^–^, which indicates that AlCl_4_^–^ will preferentially combine with AlF_3_ to form AlF_3_–AlCl_4_^–^ (Figure 2h). Therefore, both the Raman spectra and DFT calculations prove that a higher ratio of AlCl_4_^–^ exists in the electrolytes after involving inert AlF_3_ framework, facilitating the migration rate of AlCl_4_^–^ active ions.^6^ At the same time, the ratio of Al_2_Cl_7_^–^ in SSAF and F-SSAF electrolytes decreases, which can mitigate the corrosion of the Al anode by highly active Al_2_Cl_7_^–^. To understand the structure evolution of Al||C cells, the X-ray nanocomputed-tomography (XR-CT) technique is employed (Figure 2i). A continuous and compact contact of the cathode-electrolyte interface can be obviously seen by the contrast enhancement analysis, which is beneficial to inhibit graphite cathode expansion and the cycling stability. However, several technical challenges remain to be addressed, such as the limited imaging volume of high-resolution CT, the time-intensive multiangle data acquisition unsuitable for rapid dynamic processes, and the substantial computational and expertise demands for accurate reconstruction and segmentation.

### Characterizations of the Anode and Cathode Surface

**Figure 3:**
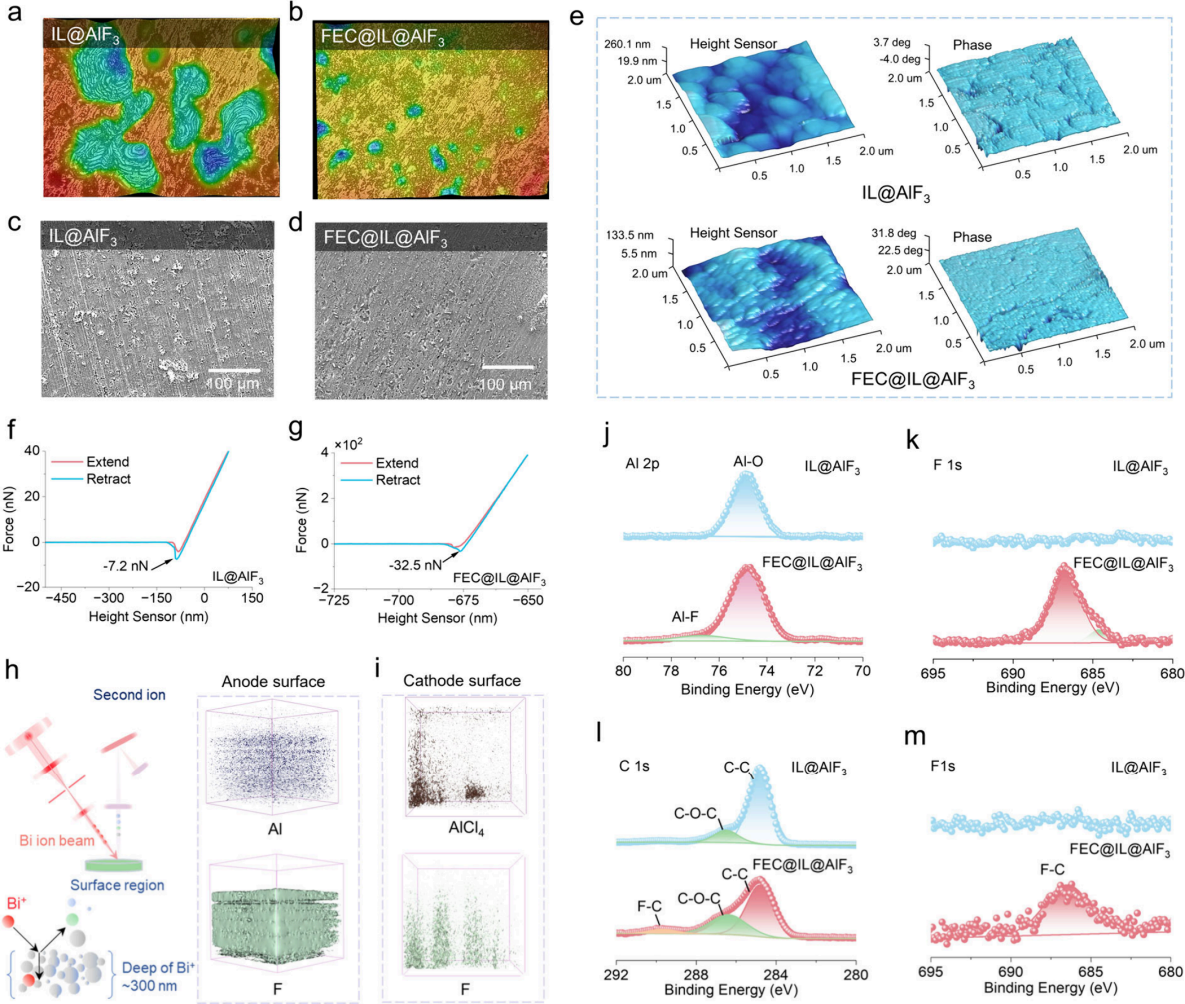
Characterizations of the anode and cathode surfaces. Optical microscopy images (a, b), SEM images (c, d), and AFM images (e) of Al anode surface after cycled in Al|SSAF|Al and Al|F-SSAF|Al cells for 40 h at 0.1 mA cm^–2^ and 0.1 mAh cm^–2^. Force–displacement curves of the deposited Al anode surface using SSAF (f) and F-SSAF (g) electrolytes. “Extend” and “retract” means the direction of motion of the cantilever with respect to the sample. ToF-SIMS 3D reconstruction of the sputtered volume of Al anode surface and schematic illustration (h) and PG cathode surface (i) using F-SSAF electrolyte. XPS measurements of Al anode surface using SSAF and F-SSAF electrolytes, with the signals Al 2p (j) and F 1s (k). XPS measurements of charged PG surface using SSAF and F-SSAF electrolytes, with the signals of C 1s (l) and F 1s (m).

AlF_3_ is an ideal framework material for stable electrolytes due to its outstanding thermal and chemical stability. These properties ensure that it remains stable in demanding electrolyte environments. The scanning electron microscopy (SEM) and Brunauer–Emmett–Teller (BET) tests display porous AlF_3_ microtubes, which possesses sufficient adsorption capacity for EMIC-AlCl_3_ (Figure S13 and S14). The framework is composed of corner-sharing AlF_6_ octahedra, forming a three-dimensional network. This network not only contributes to its structural rigidity but also provides sufficient porosity to enable excellent compatibility with EMIC-AlCl_3_, further improving ion transport properties. The Al anode surface after cycling for 40 h in Al||Al symmetrical cells at a current density of 0.1 mA cm^–2^ with a specific areal capacity of 0.1 mAh cm^–2^ is observed by optical microscope and SEM (Figures 3a-d). The F-SSAF electrolyte can mitigate large-area localized corrosion on the Al surface than the SSAF electrolyte, which proves than the FIL between Al and SSAF can form a stable surface to inhibit the nonuniform deposition of Al. The 3D measuring laser microscopy, SEM and optical microscopy images also demonstrate a more uniform Al deposition surface after the introduction of FIL and AlF_3_ (Figures S15–S17). Similarly, the surface of the cycled Al anodes using F-SSAF as electrolyte is also more homogeneous than SSAF verified by atomic force microscopy (AFM) evaluation (Figure 3e). Simultaneously, the surface average roughness (Ra) and root-mean-square roughness (Rq) of Al anode using F-SSAF are 37.8 and 30.7 nm, much lower than those of SSAF (70.5 and 55.0 nm). The force–displacement curves indicate that the surface of Al anode using F-SSAF has a strong adhesive force (Figures 3f, g), which effectively enhances the interfacial stability and promotes the rapid transport and uniform deposition of Al.^25^

To further explore the electrolyte properties in the cycled SSAIBs, the Time-of-flight secondary ion mass spectrometry (ToF-SIMS) test on both Al and C surfaces is conducted (Figures 3h,i and S18). Through the generation of 3D reconstructed imagery, the F-rich SEI and CEI films can be clearly observed, agreeing well with the X-ray photoelectron spectroscopy (XPS) surface analysis (Figure S19). The high-resolution Al 2p XPS spectra of the Al surface using F-SSAF show the presence of Al–O (74.9 eV) and Al–F (76.9 eV), but Al–F species are absent when using SSAF (Figure 3j). The high-resolution F 1s XPS spectra reveal F–C (686.8 eV) and F–Al (684.5 eV) peaks on the Al surface using F-SSAF (Figure 3k). Similar phenomenon is also observed when using EMIC-AlCl_3_ and FIL electrolytes, which further proves that the addition of FEC is the key to the formation of Al–F species (Figure S20).^27^ The detailed Cl 2p, C 1s, and O 1s XPS spectra of the four electrolytes are shown in Figure S21. On the cathode side, the C 1s and F 1s XPS spectra also confirm that the C–F (289.9 eV) and F–C (686.9 eV) peaks distribute on the carbon surface using F-SSAF (Figures 3l, m).^28^ EIS data of different cycles are consistent, also indicating the formation of stable interfacial layers (Figure S22). Therefore, the introduction of FIL into the electrodes interface proves to be conducive to the formation of F-rich SEI and CEI films, which can efficiently prevent the excessive growth of Al dendrites and improve the cycling stability.^29−32^

### All-Around Safety of SSAIBs

**Figure 4:**
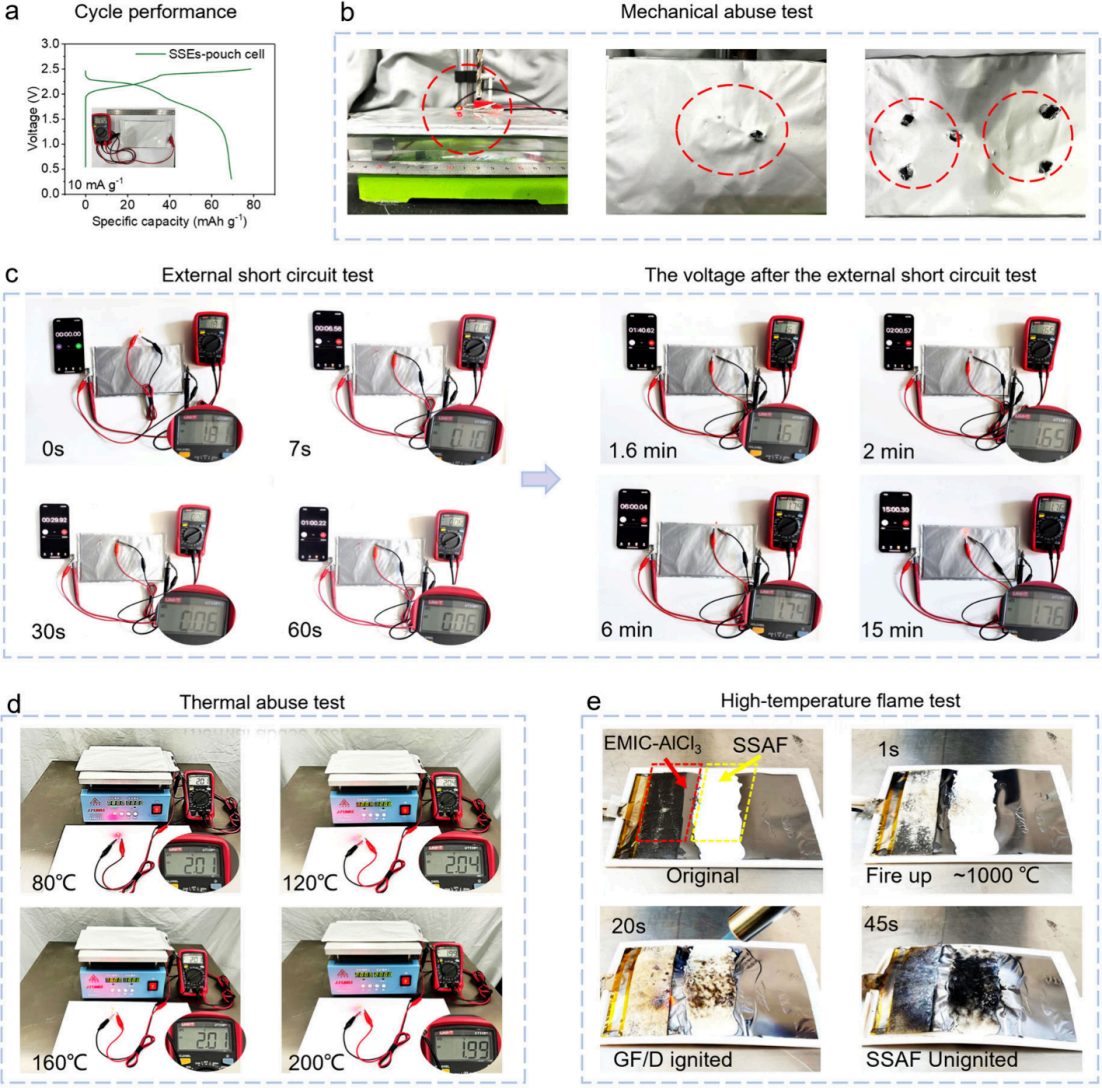
All-around safety of SSAIBs. (a) Cycling performance of pouch cells. (b) Digital photographs of pouch cells operating under harsh mechanical abuse test. (c) External short circuit test and the voltage recovery process after the external short circuit test. (d) Thermal abuse test on heating flat. (e) Digital photographs of a combustion experiment about ∼1000 °C of EMIC-AlCl_3_@GF/D and SSAF electrolytes.

To verify the practicability of F-SSAF-based SSAIBs, we assembled flexible pouch cells (Figure S23) for safety evaluation under extreme conditions. The experimental pouch cell manifests a stable charge and discharge performance (Figure 4a). In the operational state, the F-SSAF-based pouch cells under harsh mechanical abuse exhibit exceptional safety under conditions of single-point and multipoint puncture test (Figure 4b and Video S1). Even in the event of internal aluminum foil tearing, the pouch cell maintains smokeless, nonexothermic, and no electrolyte leakage, ensuring the stability of the SSAIBs during mechanical abuse (Figure S24).

An external short circuit test of the SSAIBs pouch cells at room temperature is carried out (Figure 4c). The pouch cell can light the red LED with an operational voltage of 1.81 V. After an external short-circuit test, the cell successfully lights the red LED again, and the operational voltage of the cell is recovered to 1.76 V. The thermal abuse of the pouch cell is also constructed to test the safety during a high-temperature environment (Figure 4d). The pouch cell can continuously light a red LED in the temperature range of 40 to 200 °C. Simultaneously, the open-circuit voltages of the pouch cell are measured as 2.13 2.11, 2.09, 2.05, and 2.01 V at the heat temperatures of 40, 80, 120, 160, and 200 °C, respectively (Figure S25). Furthermore, there is no excessive expansion or deformation, observed in the pouch cell throughout the entire thermal abuse process. The resistance to a high-temperature flame (approximately 1000 °C) of EMIC-AlCl_3_ and SSAF electrolytes is compared (Figure 4e and Video S2). The SSAF electrolyte still remains unignited while the EMIC-AlCl_3_@GF/D is ignited during the 45-s test period, demonstrating an excellent resistance to high-temperature after introducing the inert inorganic AlF_3_ framework.

### Recycling of AlF_3_ Inert Inorganic Framework

**Figure 5:**
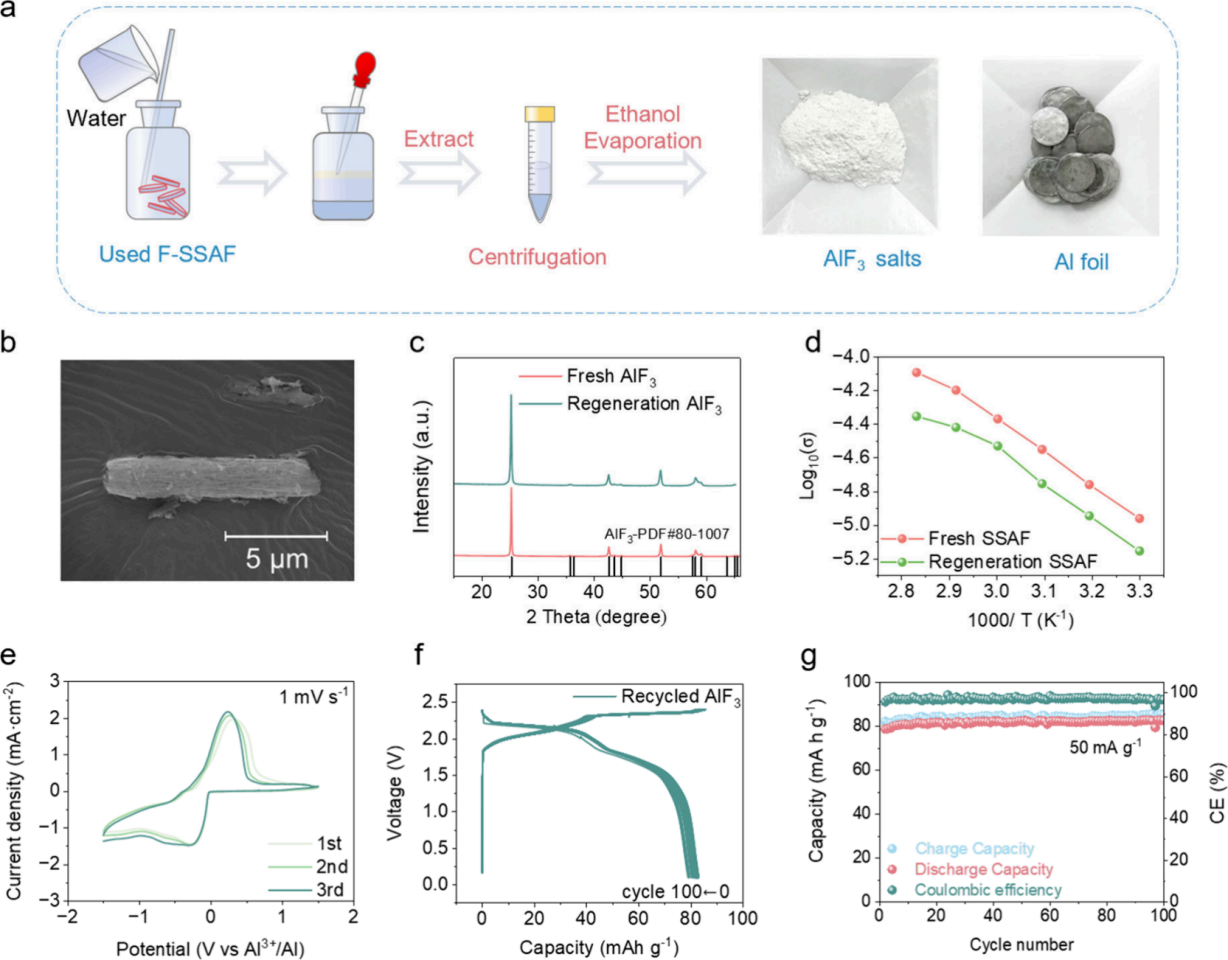
Recycling of AlF_3_ inert inorganic framework. (a) Illustration of the recycling procedures of AlF_3_ and Al foil of the used SSAF electrolyte. SEM image (b) and XRD pattern (c) of recycled AlF_3_. (d) Ionic conductivities of recycled AlF_3_ with EMIC-AlCl_3_. The electrochemical performance of SSE with recycled AlF_3_ as an inert inorganic framework in cells. (e) CV curves of the Al||Mo cells using recycled AlF_3_ with EMIC-AlCl_3_. The charge/discharge voltage profiles (f) and cycling stability (g) of Al||PG cells were obtained using recycled AlF_3_ with EMIC-AlCl_3_.

To further reduce the cost of SSAIBs, the used SSAF electrolytes were washed with deionized water, leaving white powders and Al foils after centrifugating and evaporating the ethanol solvent (Figure 5a). The obtained white powders show the SEM and X-ray diffraction pattern (XRD) as commercial AlF_3_ (Figures 5b, c), verifying the successful recycling of AlF_3_. Due to the high stability of AlF_3_ inert inorganic framework, the recycling yields of AlF_3_ from used SSAF is up to 80% (Figure S26). It is worth noting that these losses are primarily associated with small-scale laboratory experiments, where unavoidable inefficiencies are more pronounced. In large-scale industrial production, the recovery rate is expected to be higher due to improved process efficiency. In addition, Al foil can be directly used again only after a simple surface cleaning process. The recycled AlF_3_ with EMIC-AlCl_3_ exhibits similar ionic conductivities as the fresh sample (Figure 5d). Furthermore, the recycled AlF_3_ shows stable electrochemical performance when assembled in new cells (Figures 5e-g). The efficient AlF_3_ recycling would substantially lower the production cost of SSAIBs.

**Figure 6:**
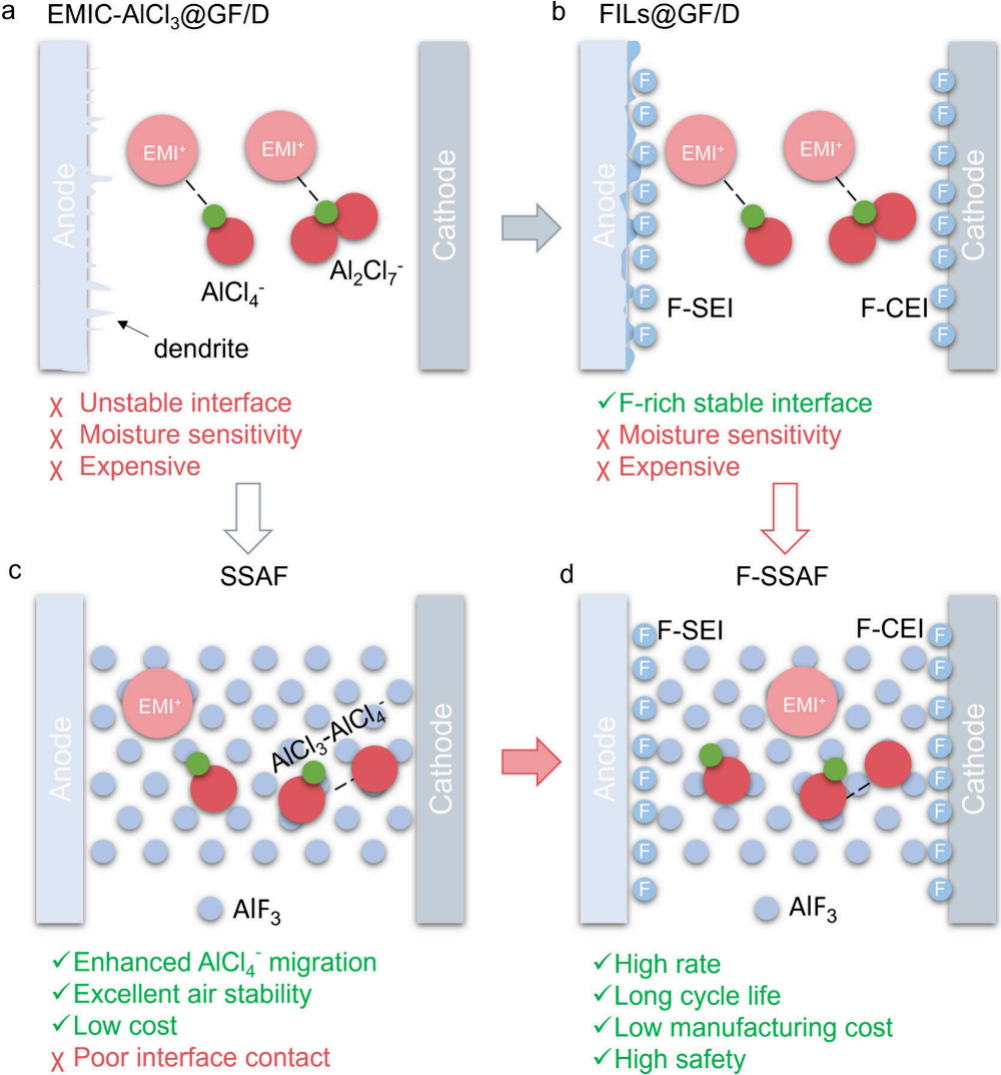
Comparison of the different electrolytes in the AIBs. Schematic illustration of the AIBs using (a) EMIC-AlCl_3_@GF/D, (b) FIL@GF/D, (c) SSAF and (d) F-SSAF electrolytes.

The comparison of the four different electrolytes (EMIC-AlCl_3_, FIL, SSAF and F-SSAF) is summarized and illustrated in Figures 6a-d. For EMIC-AlCl_3_ electrolytes, the Al deposition on the anode is nonuniform, and the interface between cathode and electrolyte is unstable, especially at high current densities, which will lead to the excessive growth of Al dendrites and affect the cycling stability^33,34^ (Figures 6a and S4a, b). To improve the stability of electrode interfaces (including anode-electrolyte and cathode-electrolyte interfaces), the FIL is introduced into the surfaces of both the anode and cathode, in situ forming F-rich SEI and CEI films. Therefore, the deposition of Al becomes more uniform, the Al dendrites can be effectively inhibited, and the cycling stability is improved (Figures 6b, S4c, d, S15 and S16). However, the disadvantages are still obvious, such as the large usage of expensive EMIC-AlCl_3_, the poor air stability and the severe corrosion of Al anode due to the large number of Al_2_Cl_7_^–^ existing in EMIC-AlCl_3_. To solve the intrinsic problems of EMIC-AlCl_3_-based nonaqueous AIBs, the SSAF electrolyte mixed with an AlF_3_ inert inorganic framework and EMIC-AlCl_3_ is constructed (Figure 6c). One the one hand, the AlF_3_ can promote the dissociation of Al_2_Cl_7_^–^ (AlCl_3_–AlCl_4_^–^) into AlCl_4_^–^, which can facilitate the migration rate of AlCl_4_^–^ active ions and enhance the ion transference number of AlCl_4_^–^. At the same time, the decrease of Al_2_Cl_7_^–^ can mitigate the corrosion of the Al anode. On the other hand, the introduction of AlF_3_ inert inorganic framework can reduce the dosage of expensive EMIC-AlCl_3_ and alleviate the moisture sensitivity of EMIC-AlCl_3_. Of course, compared with the nonaqueous AIBs, the SSAF-based SSAIBs have the features of no leakage risk, high safety, inhibition of graphite cathode expansion but poor contact interface between electrodes and electrolyte. Finally, the F-SSAF taking advantage of both FIL and SSAF is built, and high-performance SSAIBs are achieved (Figure 6d, Table S4 and S6).

## Conclusions

By introducing an AlF_3_ inert inorganic framework, we successfully design solid-state aluminum-ion batteries with long cycle life and low manufacturing cost. The dissociation of Al_2_Cl_7_^–^ into AlCl_4_^–^ is promoted by AlF_3_, thus resulting in more AlCl_4_^–^ active ions and less Al_2_Cl_7_^–^ in SSAF. This will lead to high ionic conductivity (7.0 mS cm^–1^), high anion-ion transference number (0.50), and relaxative Al anode corrosion. The dilution effect of AlF_3_ can reduce the dosage of expensive EMIC-AlCl_3_ and the moisture sensitivity of EMIC-AlCl_3_ is also improved. The in situ formed F-rich SEI and CEI films at the anode-electrolyte and cathode-electrolyte interfaces further uniform the Al deposition/dissolution and therefore inhibit the growth of Al dendrite. The F-SSAF-based symmetric cells exhibit ultralong stable deposition and dissolution of Al up to 4000 h. Meanwhile, the full cells deliver an ultralong-life (10000 cycles) with an average Coulombic efficiency of >99%. In addition, the recyclability of AlF_3_ further reduces the production costs of SSAIBs. As a new concept, there is still much to explore, such as the study for other inert inorganic frameworks with better interface and low cost, but our findings open a way to practical SSAIBs.

## Data Availability

All relevant data that support the findings of this study are presented in the manuscript and Supporting Information. Source data are available from the corresponding author upon reasonable request.

## Supporting Information Available

Experimental details, materials and methods, characterizations, DFT calculation, supplementary figures and tables (PDF)
Video S1. The F-SSAF-based pouch cells under harsh mechanical abuse (MP4)
Video S2. The F-SSAF electrolyte (left, white) and IL@GF/D (right) under a high-temperature flame (approximately 1000 °C) (MP4)
Transparent Peer Review report available (PDF)
