# Supplementary material for: A Recyclable Inert Inorganic Framework Assisted Solid-State Electrolyte for Long-Life Aluminum Ion Batteries
Source: ACS Cent Sci. 2024 Dec 19;11(2):239–47. doi: 10.1021/acscentsci.4c01615 (PMC11869131; doi:10.1021/acscentsci.4c01615)
Supplement: Supplementary file 1 — oc4c01615_si_001.pdf [file oc4c01615_si_001.pdf]

Supplementary Information

A recyclable inert inorganic framework assisted solid-state electrolyte for long-life aluminum ion batteries

Ke Guo[1], Wei Wang[2*], Wei-Li Song[1], Shijie Li[2], Xueyan Du[3] and Shuqiang Jiao[1,2,3*]

[1] Institute of Advanced Structural Technology, Beijing Institute of Technology, Beijing 100081, China.

[2] State Key Laboratory of Advanced Metallurgy, University of Science and Technology Beijing, Beijing 100083, China.

[3] State Key Laboratory of Advanced Processing and Recycling of Nonferrous Metals, Lanzhou University of Technology, Lanzhou 730050, China.

*Corresponding author. E-mail: wwang@ustb.edu.cn; sjiao@ustb.edu.cn

Experimentals Sections

Materials and methods.

Aluminum foil (100 μm thickness, 99.99%), molybdenum foil (20 μm thickness, 99%), Aluminum chloride ($AlCl_3$, anhydrous, >99%, Macklin), 1-ethyl-3-methylimidazolium chloride (EMIC, $C_6H_{11}ClN_2$, >98.0%, Macklin), aluminum fluoride (AlF_3, 99.9% metals basis, Macklin), fluoroethylene carbonate (FEC, $C_3H_3FO_3$, 99%, Meryer), graphite (99.95% metals basis, 4000 mesh, Shanghai Aladdin Biochemical Technology Co., Ltd.) were obtained from commercial sources.

Preparation of EMIC-$AlCl_3$, FIL and SSAF.

The EMIC and $AlCl_3$ was mixed with a molar ratio 1:1.5. Then, the EMIC-$AlCl_3$ was stirring at least 24 h. The EMIC-$AlCl_3$=1:1.5(molar ratio) were mixed under constantly stirring in an argon-atmosphere glovebox. Different volumes (1 *vol*%, 2 *vol*%, and 3 *vol*%) of FEC were dissolved into the EMIC-$AlCl_3$ solution under stirring to obtain a FIL. Then the FIL was infiltrated into the Al foil and graphite electrode surfaces, respectively, to prepare the cell assembly. Furthermore, different amounts (45 *wt*%, 50 *wt*%, and 55 *wt*%) of the AlF_3 were mixed with the EMIC-$AlCl_3$ solution, and then SSAF was obtained after standing still for 24 h.

Assembly of the SSEs AIBs.

PG was provided by JiangXi Dasen Technology Co., Ltd. Generally, 4000 mesh graphite powders were mixed with carbon black, polyvinylidene fluoride (PVDF) powder in a ratio of 7:2:1. Then the graphite viscous solution was coated onto the Mo foil and dried in vacuum at 80 °C. The SSAF

electrolyte was compressed into a 14 mm diameter disc and the cells were assembled in a Swagelok-type cell. The assembly process then involved sequentially assembling FIL@Aluminum foil, SSAF electrolytes, and FIL@cathode material into the Swagelok-type cell.

Materials characterizations.

The Al surface morphologies were characterized by an optical microscope (Keyence Corporation). 3D Measuring Laser Microscope (OLS5000, Olympus Corporation) and Scanning Electron Microscopy (SEM, ZEISS GeminiSEM 300). AFM characterization was carried out on a Bruker Dimension Fastscan instrument. ToF-SIMS (PHI nano ToF II, ULVAC-PHI, Inc. Japan) was investigated surface components on Al and cathode surface. X-ray photoelectron spectra (XPS) were conducted to investigate the surface components on Al deposition and charged cathode surface. X-ray source: Al Kα (Al target, 1486.8 eV, line width 0.6 eV). Charge correction: C 1s 284.8 eV. Raman spectra of electrolytes were obtained with a laser wavelength of 325 nm in a frequency range from 50 to 4000 cm^{-1} on HORIBA Scientific LabRAM HR Evolution. The Brunauer-Emmett-Teller (BET, Quantachrome Nova 2000e) method was used to estimate the specific surface area and pore diameter distribution of AlF_3 powder. XR-CT experiments were performed using an Xradia 520 Versa system (Zeiss). To achieve sufficient resolution, a customized Swagelok cell with a small diameter was designed and fabricated. This cell ensured both gas impermeability and a clear density contrast between the electrolyte and the Swagelok (PTFE material). The density difference was essential for effectively distinguishing the solid-state electrolyte framework from the Swagelok components during

image analysis. The CT system acquired X-ray projection data from multiple angles, which was then processed via computational reconstruction to generate cross-sectional and 3D images.

Theoretical calculations.

DFT calculations were performed using the Gaussian 16 software Package [1]. The geometry structures were fully optimized at the level of B3LYp/def2-Svp with Grimme's DFT-D3(BJ) empirical dispersion correction to describe the weak interaction [2, 3]. Vibration frequencies calculation were also carried out at the same level to confirm that the optimized structure is the local minimum point on the potential energy surface. The adsorption energy (Δ E) of AlX_3 (X=F, Cl) combined with $AlCl_4^-$ and $Al_2Cl_7^-$ were calculated using following Eq. 1:

$$\Delta E = E_{AlX_3} - (E_{AlX_3} + E_{ion}) (1)$$

Characterization of electrochemical performance.

All configurations of Swagelok-type cells were assembled in an argon-filled glove box (H_2O < 0.1 ppm, O_2 < 0.1 ppm). The electrochemical stability of the electrolytes was investigated by LSV on EC-LAB test. The deposition and dissolution curves were collected with a battery-test system (EC-LAB) at a scan rate of 5 mV s^{-1}. Galvanostatic charge/discharge measurement was conducted on the multichannel battery test system (NEWARE). The ionic conductivity of electrolytes were measured by EIS at the frequency range of 100 kHz to 0.1 Hz with a perturbation signal of 10 mV under the open circuit potential by an EC-LAB test from 30 to 80 °C in a constant temperature oven (DGH-9070A). Before the conductivity measurements, the Mo|Electrolyte|Mo symmetric cells were still all

night to form a stable contact and kept at each test temperature (from 30 to 80 °C) for 1.0 h to reach thermal equilibrium in a constant temperature oven. The ionic conductivity (σ) was calculated according to the Eq.2:

$$\sigma = \frac{L}{S \times R}$$

where L (cm) represents the thickness of the SSE, S (cm^2) the area of the electrolyte, and R (Ω) the bulk ohmic resistance obtained by EIS, respectively

The ion transference number of the electrolyte was obtained using a combining of EIS and DC polarization techniques in Al|Electrolyte|Al symmetrical Swagelok-type cell. The EIS data were analysed using ZView fitting software The transference number t_- was calculated on the basis of Eq. 3:

$$t_- = \frac{I_s(\Delta V - I_o R_o)}{I_o(\Delta V - I_s R_s)} \quad (3)$$

where ΔV is the applied potential; I_o and I_s are the initial and steady-state current, and R_o and R_s are the initial and steady-state resistance, respectively.

Supplementary Figures

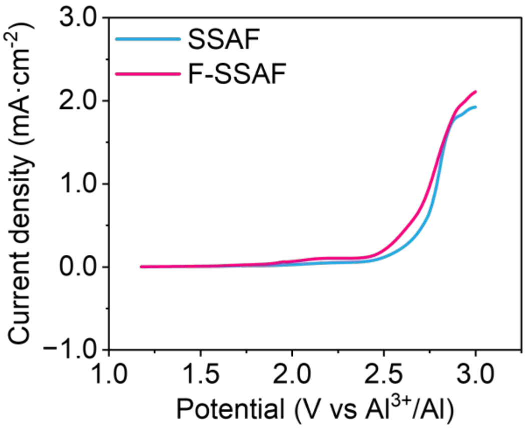

Figure S1. LSV curves of the Al||Mo cells using SSAF and F-SSAF electrolytes.

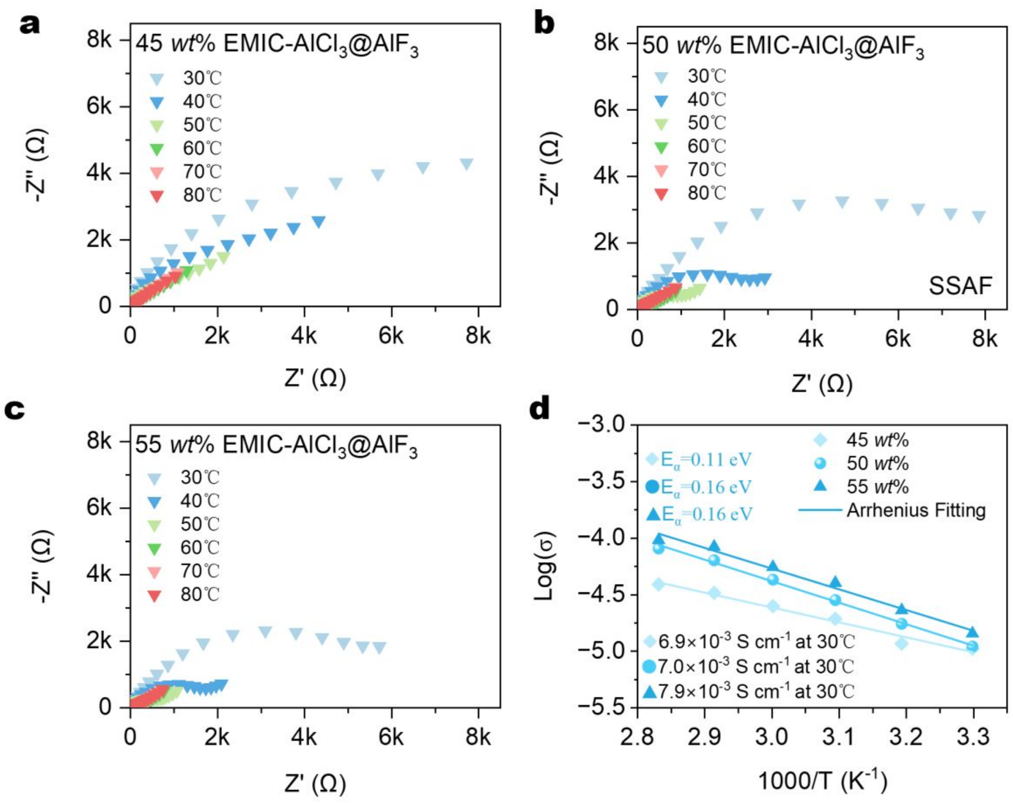

Figure S2. EIS curves and Arrhenius activation energy of SSAF electrolyte.

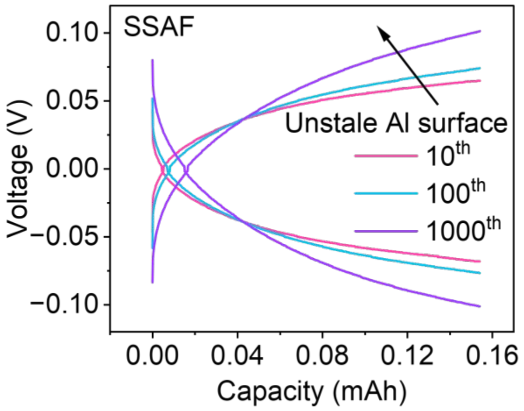

Figure S3. The voltage profiles (10th, 100th, 1000th cycle) of the Al|SSAF|Al symmetric cell.

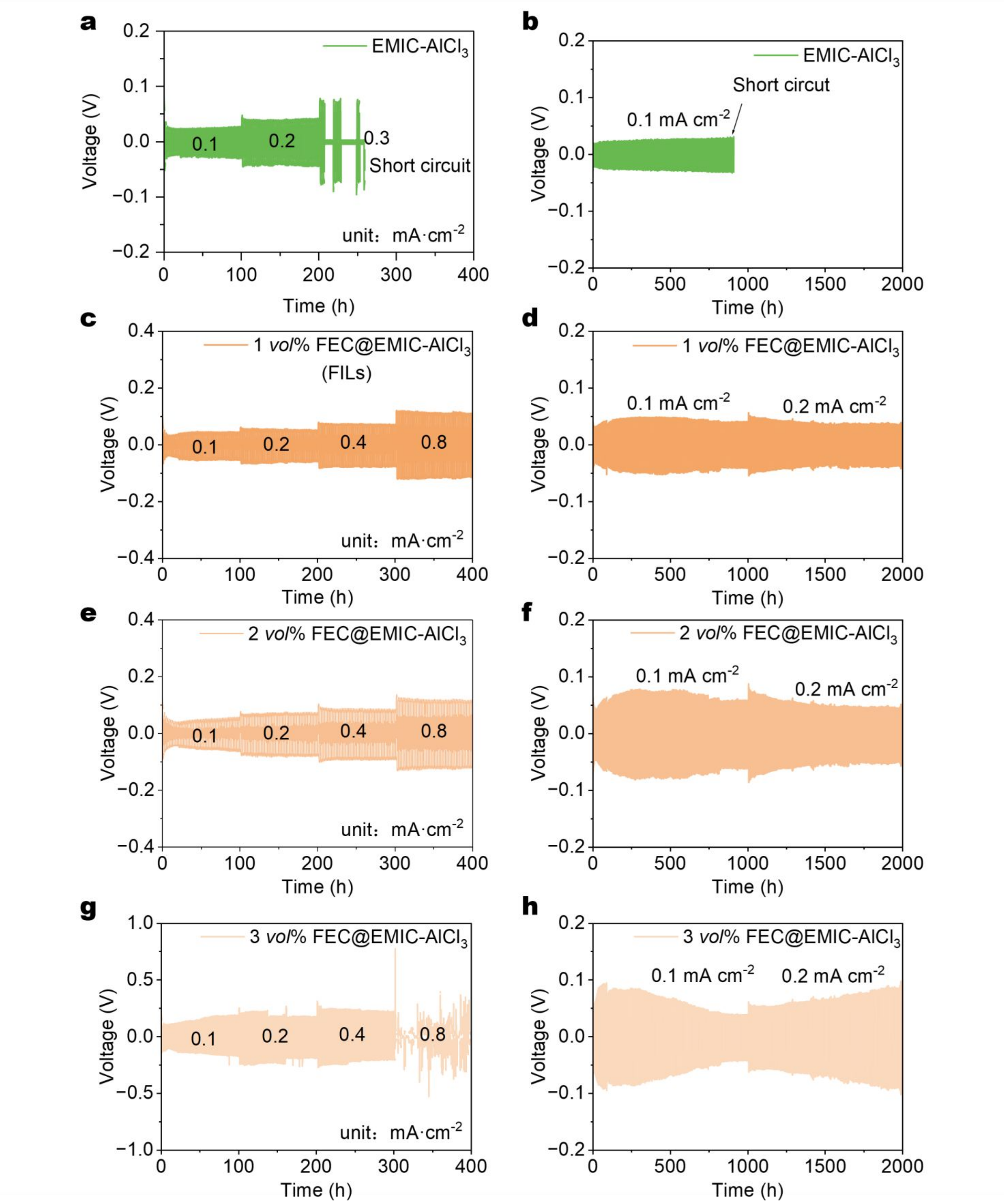

Figure S4. Comparison of Al deposition/dissolution cycling processes for Al|EMIC-$AlCl_3$|Al and Al|FEC(1 *vol*%, 2 *vol*% and 3 *vol*%)@EMIC-$AlCl_3$|Al symmetric cells at different current densities.

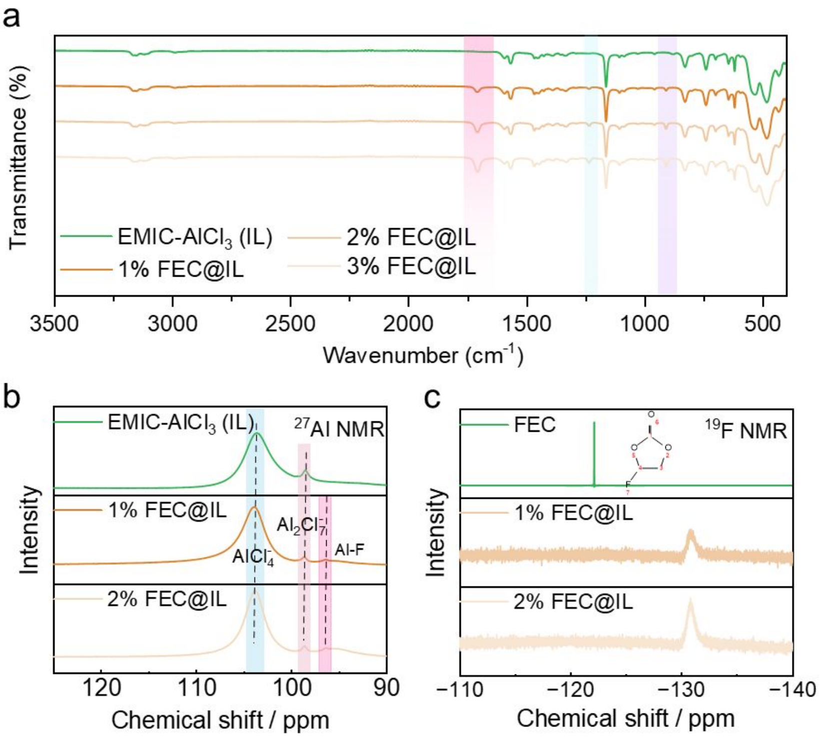

Figure S5. (a) FT–IR spectra of EMIC-$AlCl_3$ (IL), 1% FEC@IL, 2% FEC@IL and 3% FEC@IL; (b) ^{27}Al NMR spectra of EMIC-$AlCl_3$ (IL), 1% FEC@IL and 2% FEC@IL; (c) ^{19}F NMR of FEC, 1% FEC@IL and 2% FEC@IL.

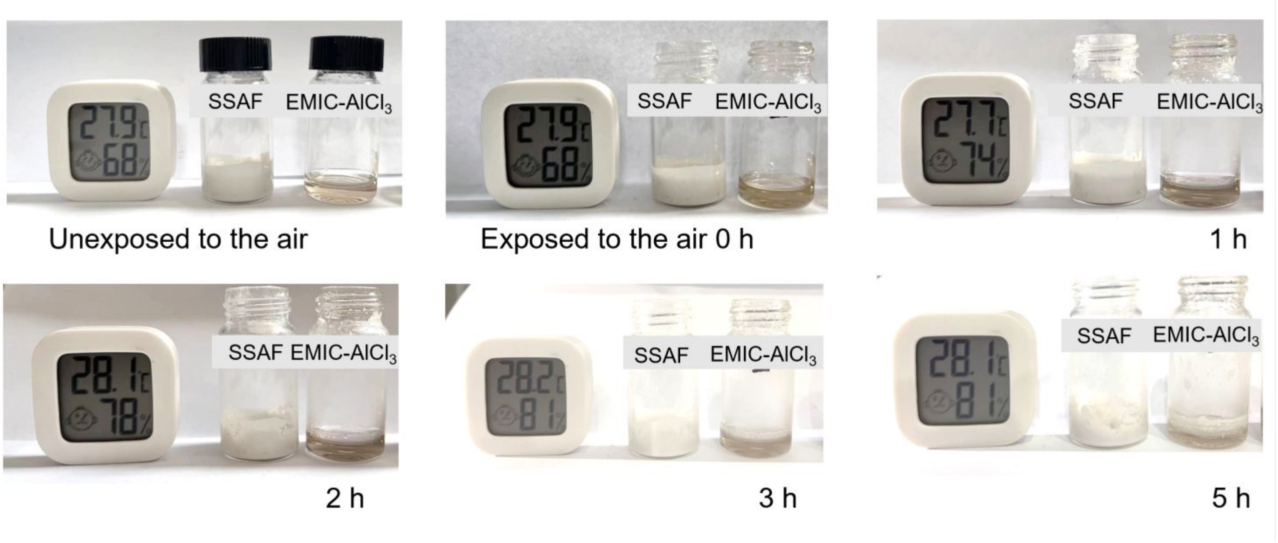

Figure S6. Air stability test of SSAF and EMIC-$AlCl_3$ electrolytes.

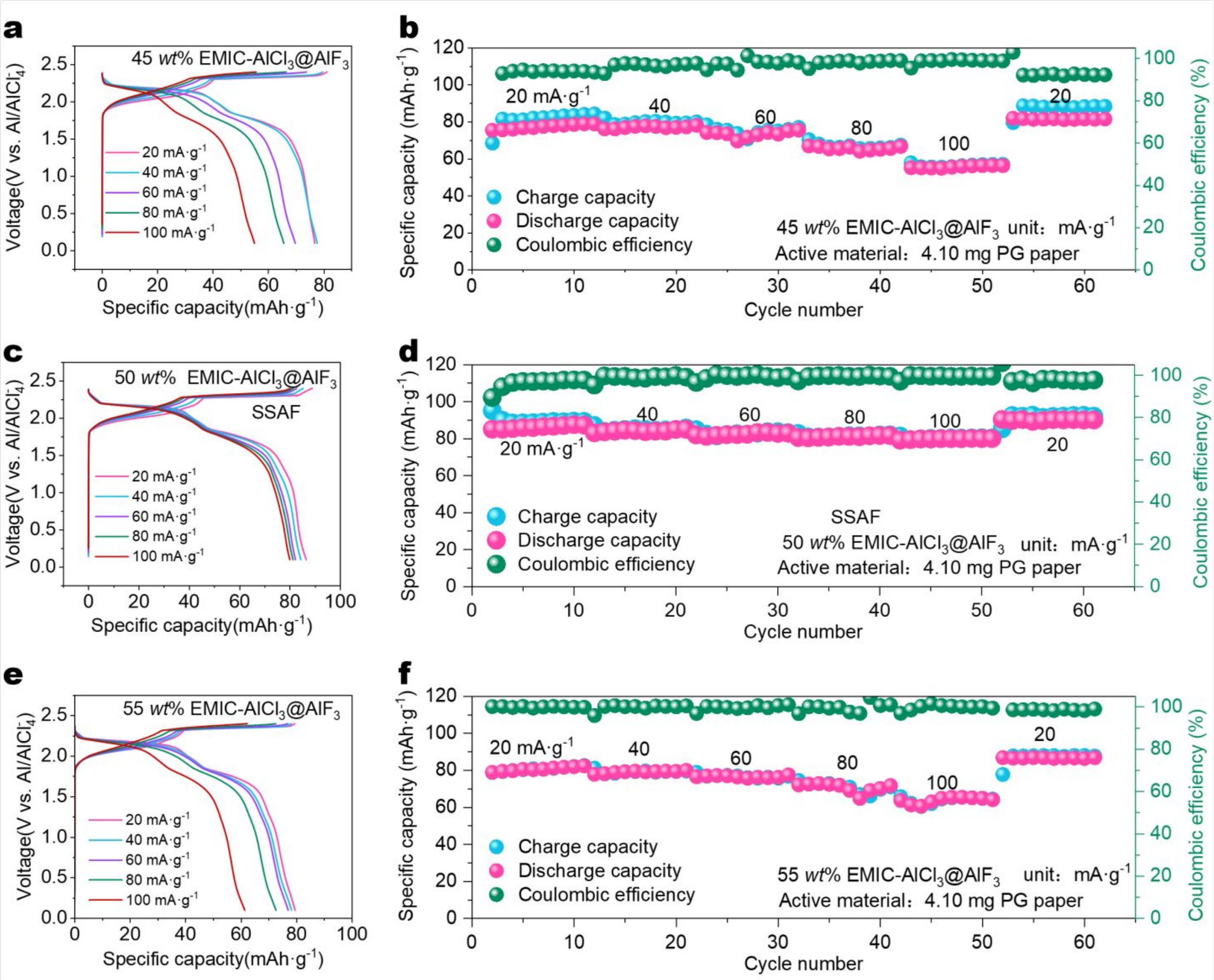

Figure S7. Comparison of the rate performance of SSAF electrolytes with different EMIC-$AlCl_3$ ratios (45 *wt*%, 50 *wt*%, 55 *wt*%).

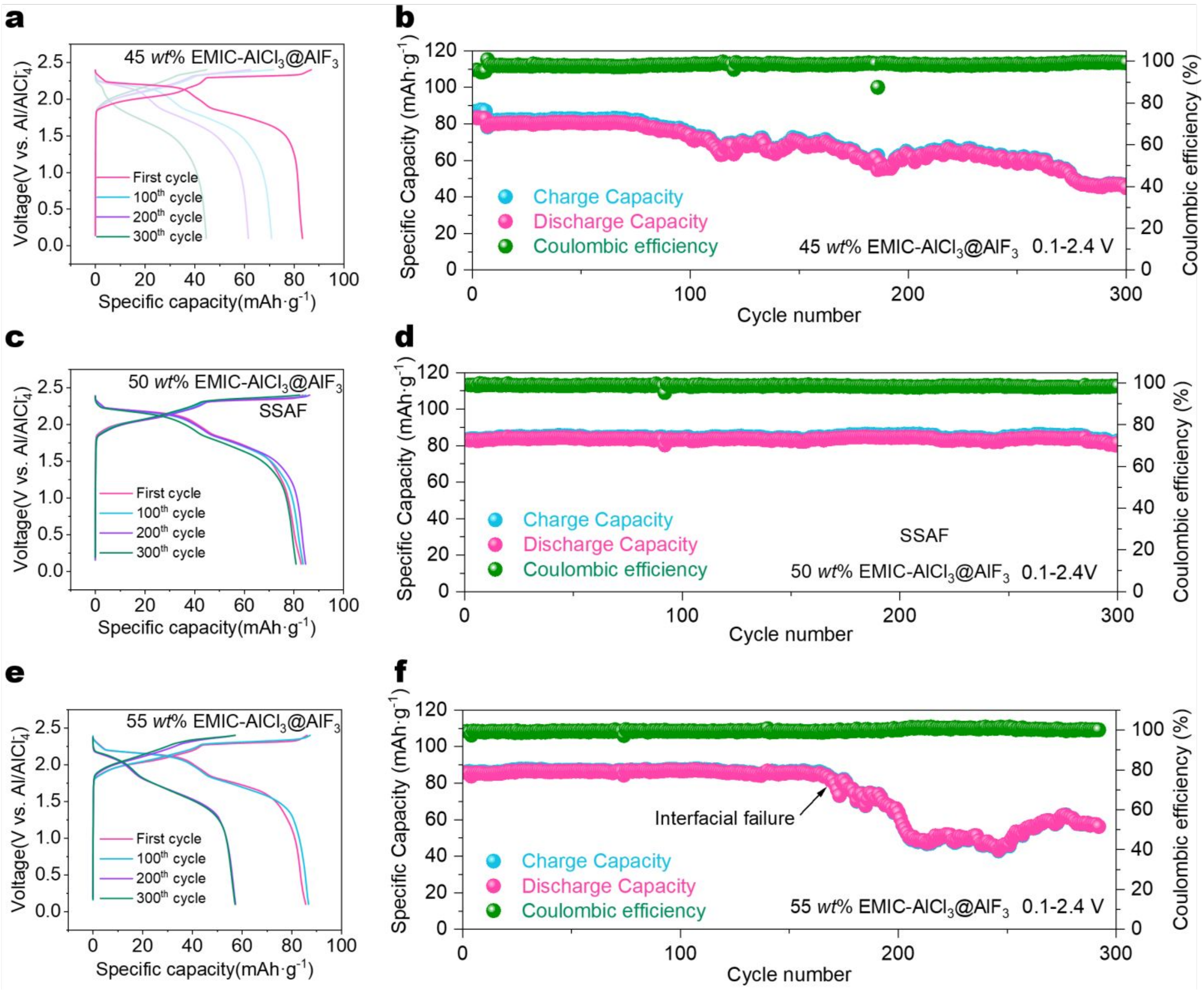

Figure S8. Comparison of the charge/discharge voltage profiles and long cycling stability (300 cycle at 50 mA g^{-1}) of SSAF electrolytes with different EMIC-$AlCl_3$ ratios (45 *wt*%, 50 *wt*%, 55 *wt*%).

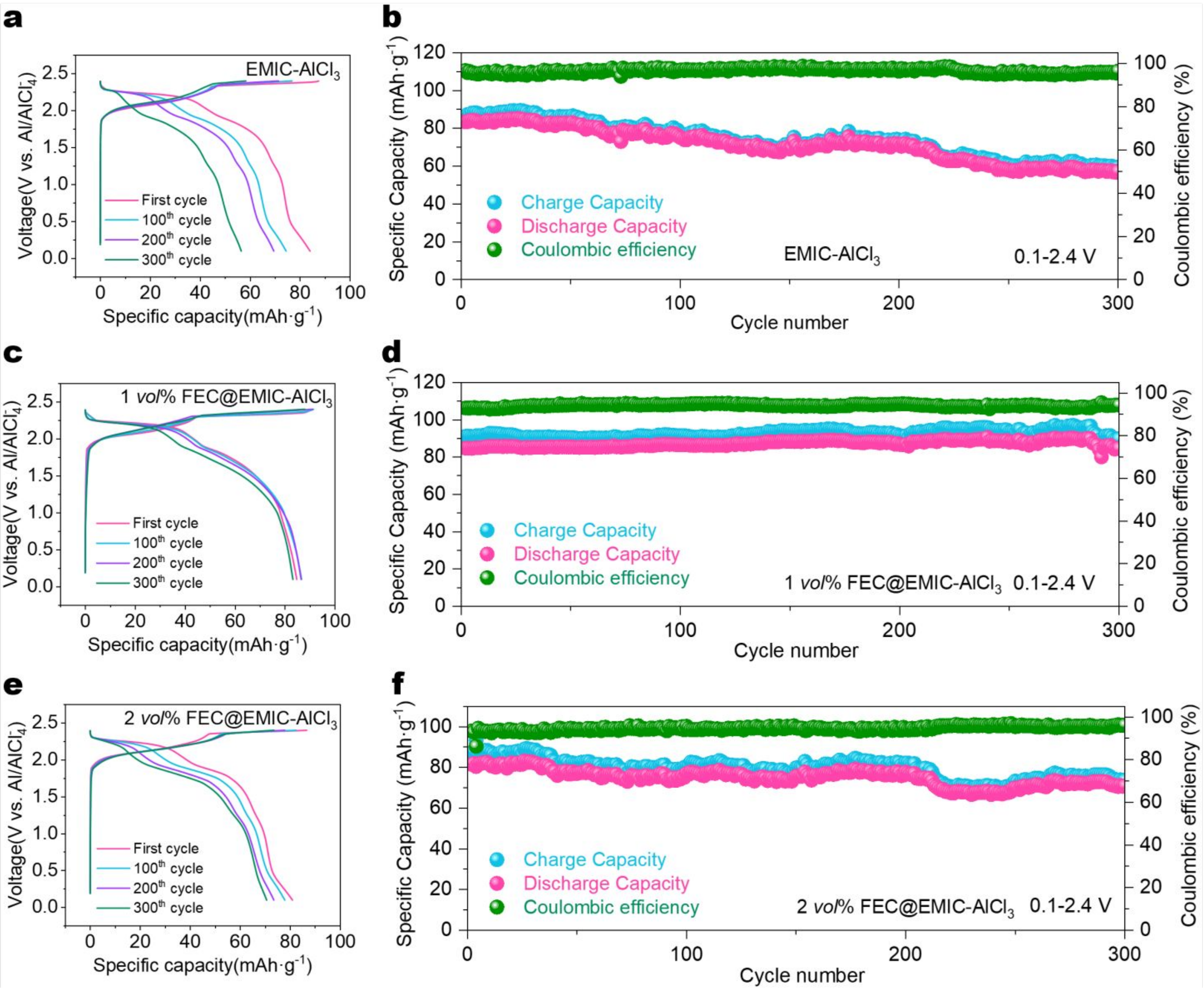

Figure S9. Comparison of the charge/discharge voltage profiles and long cycling stability (300 cycle at 50 mA g^{-1}) of EMIC-$AlCl_3$ and FEC (1 *vol*% and 2 *vol*%)@EMIC-$AlCl_3$ electrolytes.

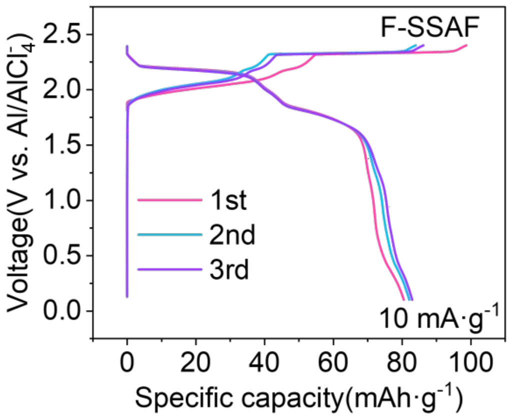

Figure S10. The charge/discharge voltage profiles of Al|F-SSAF|PG cells at a small current density of 10 mA g^{-1}.

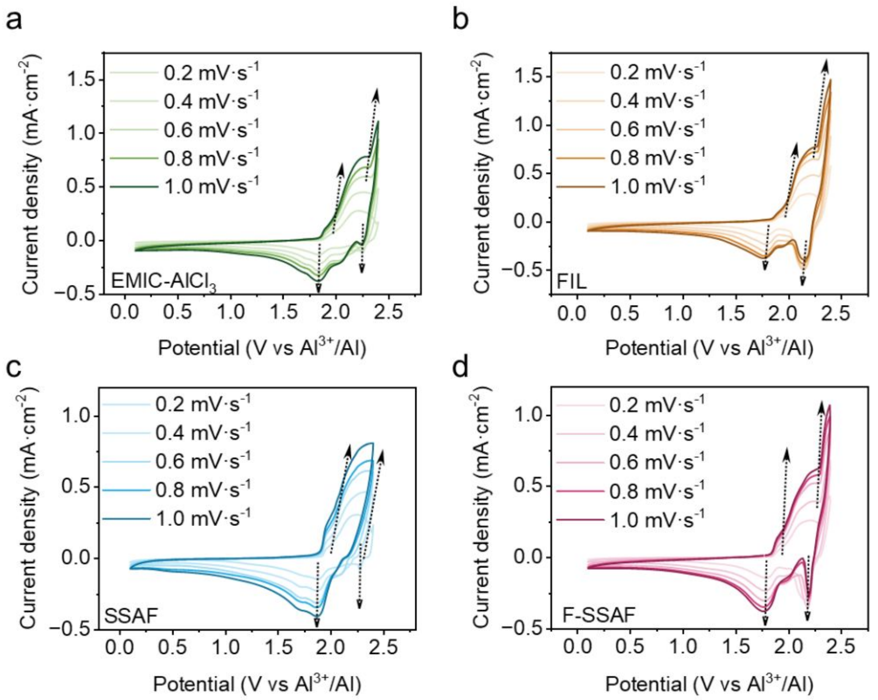

Figure 11. CV curves of the EMIC-$AlCl_3$, FIL, SSAF and F-SSAF at 0.2, 0.4, 0.6 0.8, 1.0 mV s^{-1}.

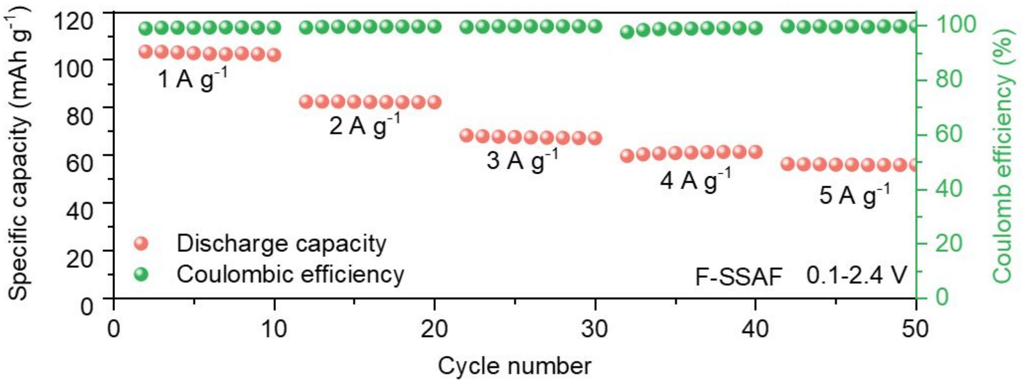

Figure S12. Rate performance of F-SSAF with graphite cathode at various current densities from 1 to 5 A g^{-1}.

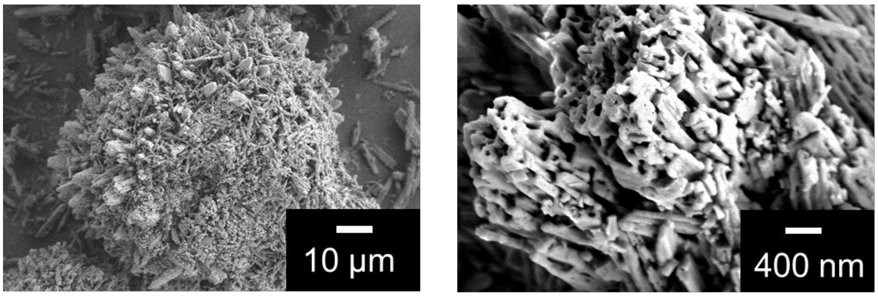

Figure S13. The SEM images of AlF_3.

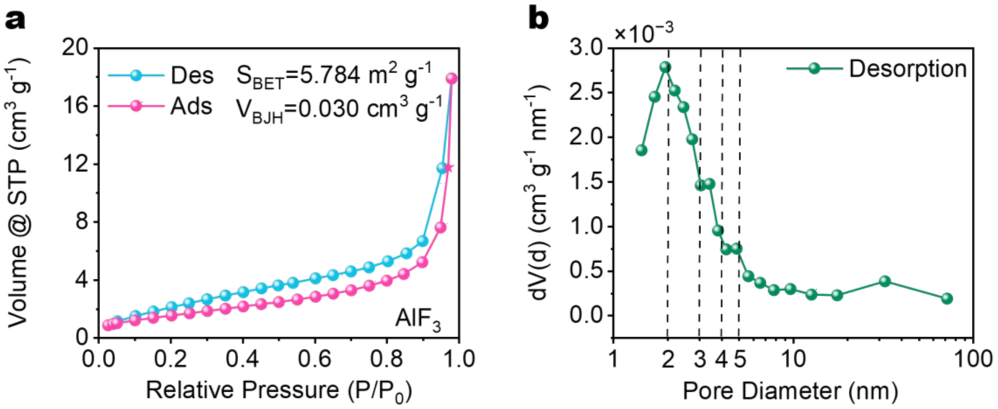

Figure S14. The BET tests of AlF_3.

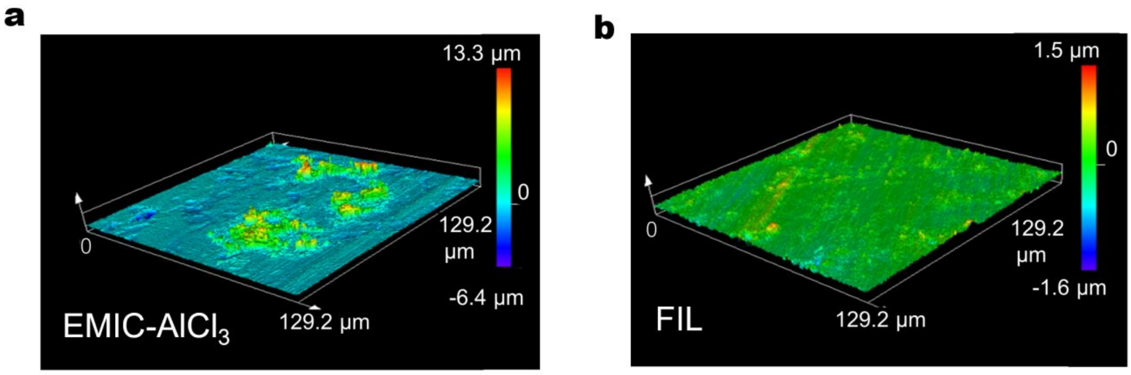

Figure S15. 3D Measuring Laser Microscope images of Al anode surface after cycled in Al|EMIC-$AlCl_3$|Al (a) and Al|FIL|Al (b) symmetric cells for 40 h at 0.1 mA cm^{-2}.

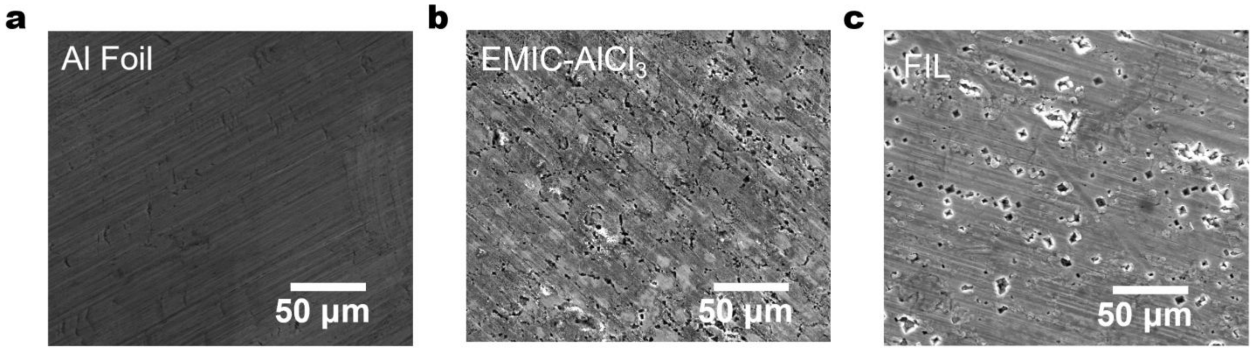

Figure S16. SEM images of pristine Al surface (a), and Al anode surface after cycled in Al|EMIC-$AlCl_3$|Al (b) and Al|FIL|Al (c) symmetric cells.

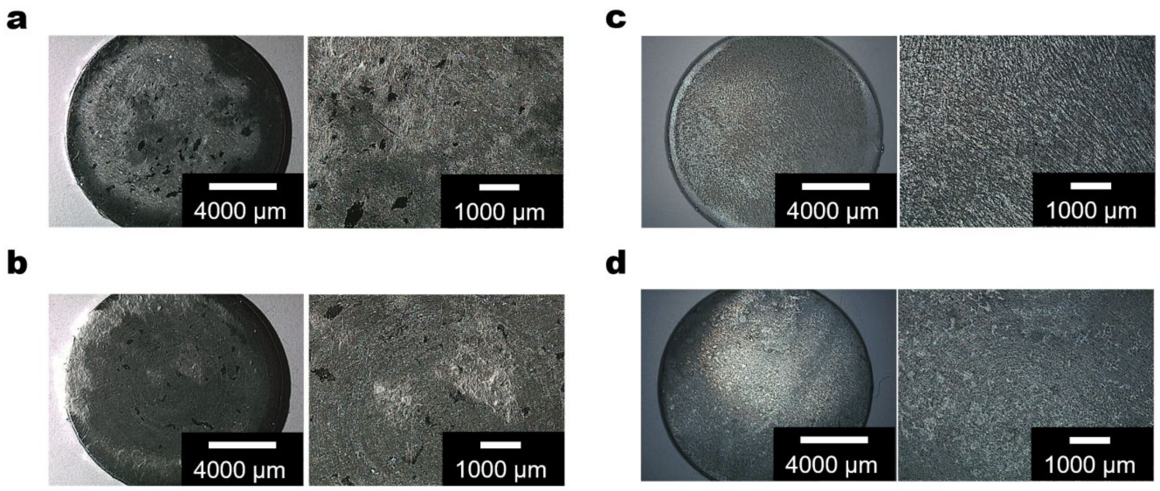

Figure S17. Optical microscopy images of Al anode surface after cycling with different electrolytes. (a) EMIC-$AlCl_3$, (b) FIL, (c) SSAF and (d) F-SSAF.

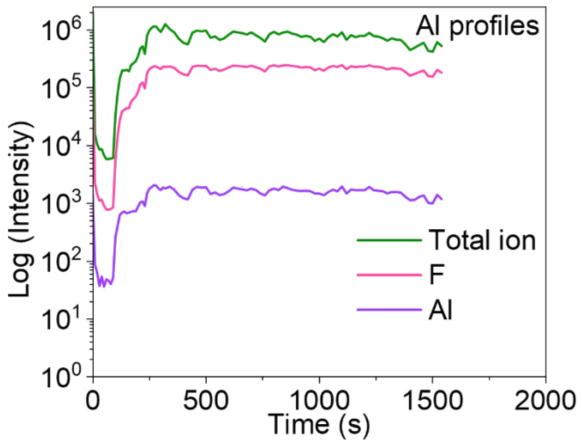

Figure S18. Depth profiles of the Al anode surface after cycling with F-SSAF electrolyte.

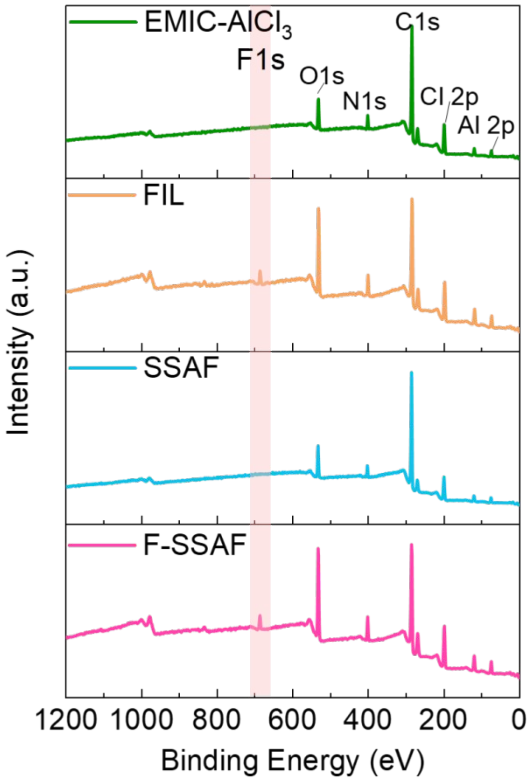

Figure S19. XPS full spectra of Al anode surface after cycling with EMIC-$AlCl_3$, FIL, SSAF and F-SSAF electrolytes.

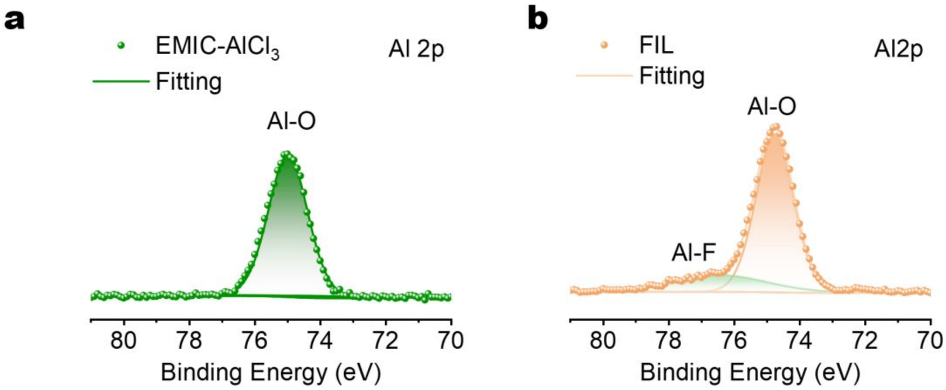

Figure S20. Al 2p XPS spectra of Al surface after cycling with (a) EMIC-$AlCl_3$ and (b) FIL electrolytes.

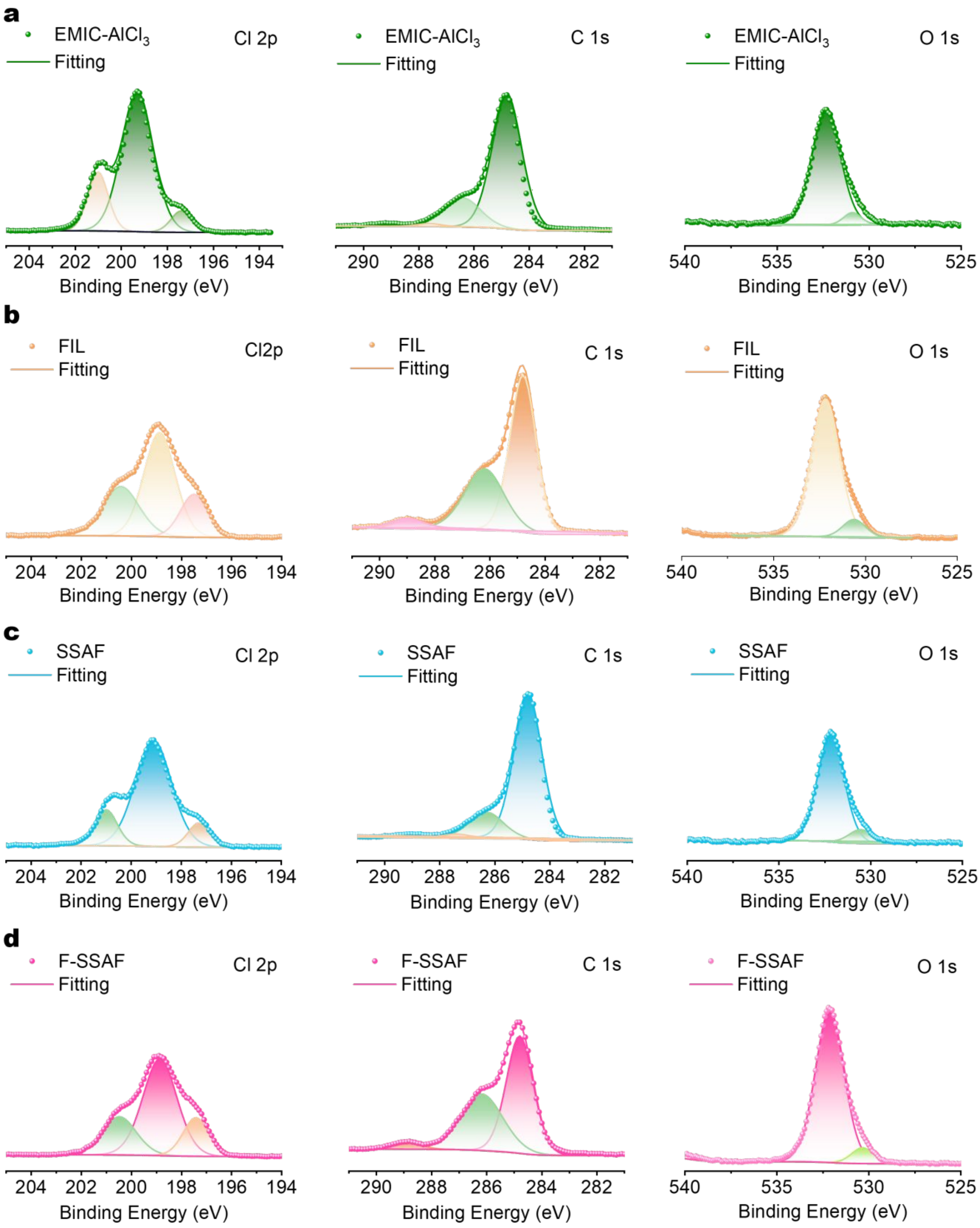

Figure S21. The Cl 2p, C 1s, and O 1s XPS spectra of Al surface after cycling with (a) EMIC-$AlCl_3$, (b) FIL, (c) SSAF and (d) F-SSAF electrolytes.

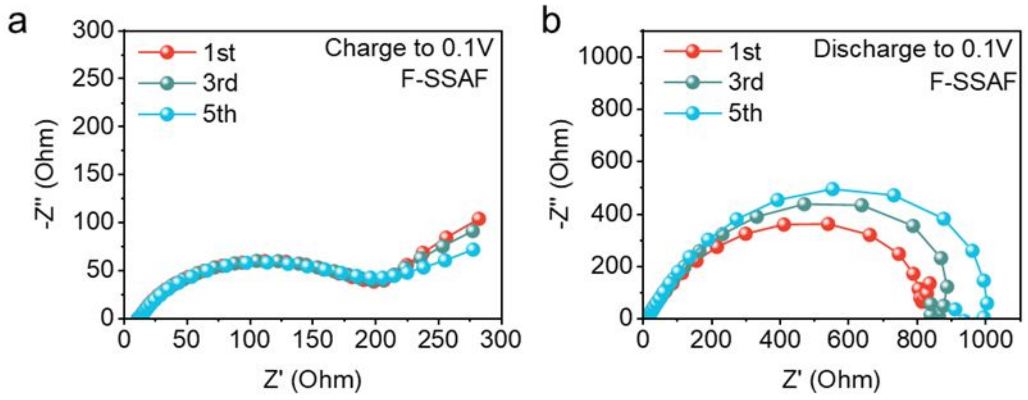

Figure S22. (a) Nyquist plots of Al|F-SSAF|PG cell charged to 2.4V after different cycles; (b) Nyquist plots of Al|F-SSAF|PG cell discharged to 0.1V after different cycles.

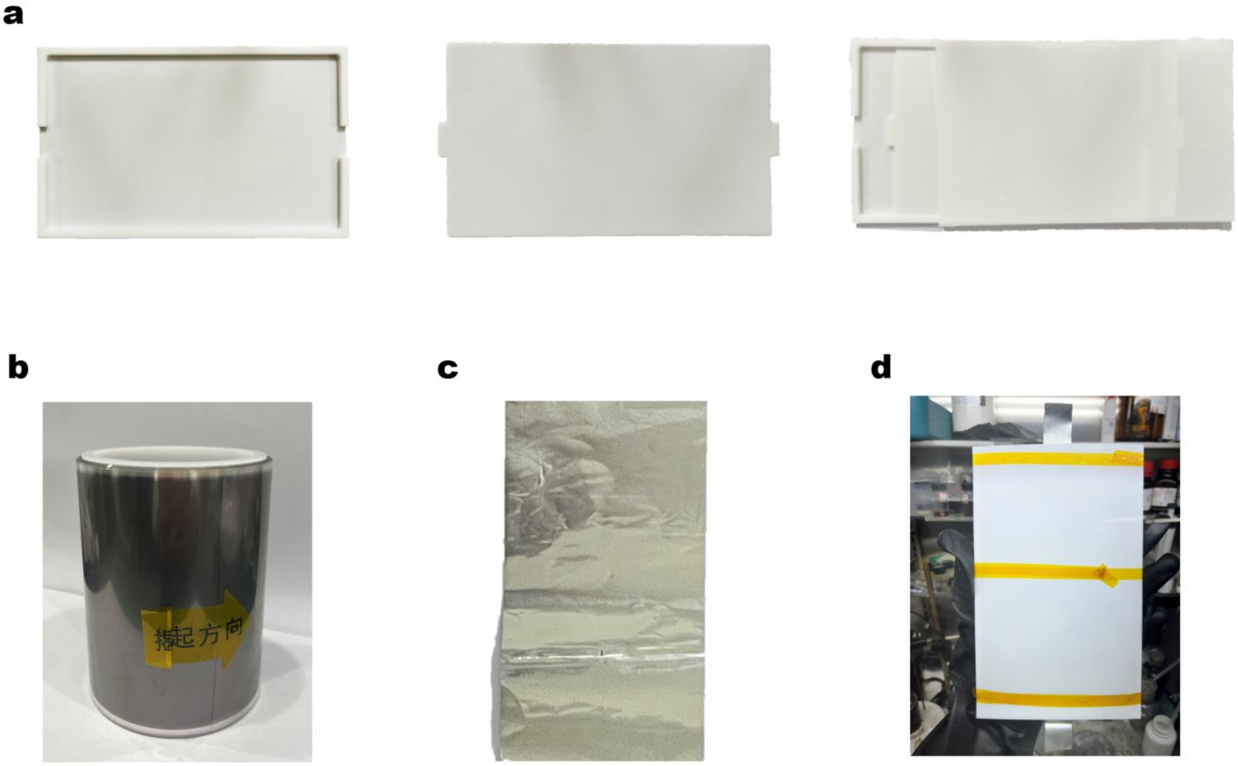

Figure S23. The assembly of pouch cell. (a) Cover assembly. (b) Cathode material. (c) Aluminum foil. (d) The assembled SSAIBs.

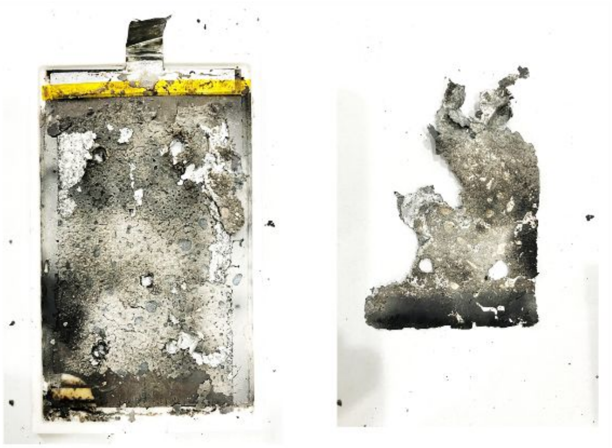

Figure S24. Inner cell after mechanical abuse.

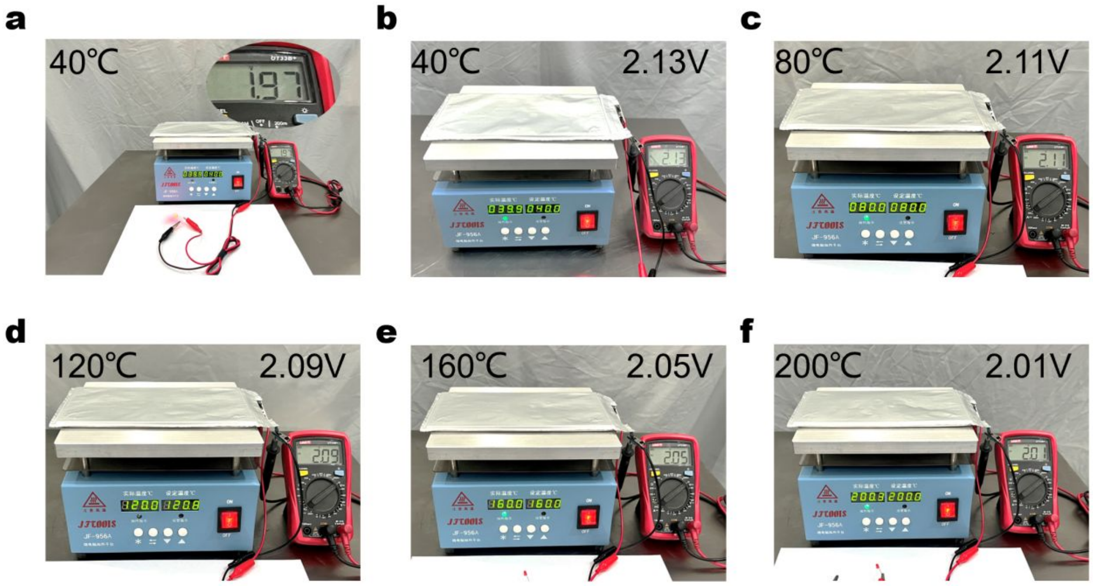

Figure S25. (a) The working voltages of the pouch cell at the heat temperatures of 40 ℃. (b-f) The open-circuit voltages of the pouch cell at the heat temperatures of 40, 80, 120, 160, and 200 ℃, respectively.

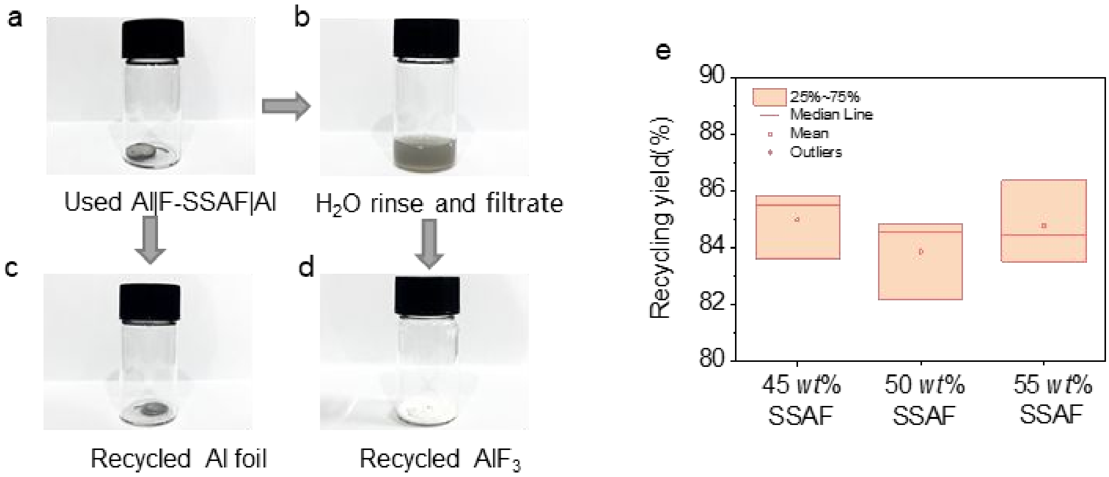

Figure S26. (a-d) Recycling procedure of AlF_3 and Al foil from Al|SSAF|Al c ell. (e) Recycling yields of AlF_3 from used SSAF with different EMIC-$AlCl_3$ ratios (45 wt%, 50 wt%, 55 wt%)

Supplementary Tables

Table S1. Ionic conductivity of SSAF electrolytes with different EMIC-$AlCl_3$ ratios.

Temperature (℃)	σ (mS cm^{-1})		
	45 *wt*% EMIC-$AlCl_3$	50 *wt*% EMIC-$AlCl_3$	55 *wt*% EMIC-$AlCl_3$
30	6.882	7.017	7.872
40	7.204	8.579	9.670
50	8.967	10.580	12.317
60	10.024	12.672	14.162
70	11.273	15.039	16.948
80	12.148	16.696	17.966

Table S2. Ionic conductivity of F-SSAF electrolytes with different FEC (1 *vol*%, 2 *vol*% and 3 *vol*%)@EMIC-$AlCl_3$.

Temperature (℃)	σ (mS cm^{-1})		
	1 *vol*% FEC@ EMIC-$AlCl_3$	2 *vol*% FEC@ EMIC-$AlCl_3$	3 *vol*% FEC@ EMIC-$AlCl_3$
30	6.464	6.155	5.786
40	8.013	7.532	7.128
50	9.622	9.042	8.632
60	11.621	10.458	10.794
70	12.836	11.607	12.053
80	14.788	12.627	12.875

Table S3. Computational details of the ion transference number.

Parameters	EMIC-$AlCl_3$	FIL	SSAF	Unit
Ion transference number (t)	0.139	0.237	0.504	/
Polarization voltage (ΔV)	10	10	10	mV
Initial current (I_o)	10.33	13.10	10.62	μ A
Steady state current (I_s)	1.58	3.00	3.03	μ A
Initial interfacial resistance (R_o)	661.35	946.59	416.76	Ω
Steady-state interfacial resistance (R_s)	4130.67	4105.93	2258.4	Ω

Table S4. Comparison of different electrolytes.

Electrolyte	Ion transference number	Ion conductivity mS cm^{-1}	The working time	Ref.
**F-SSAF**	**0.50**	**7.0**	**4000 h**	**This work**
PA-EMIC-based -GPE	/	6.61	800 h	4
EMIC-based GPE	0.019	5.57	/	5
PA-Et_3NHCl-based GPE	/	3.86	3800 h	6
PA-EMIC-based GPE	/	1.66	/	7
EA-EMIC-based GPE	0.171	1.39	/	8
Acrylamide Et_3NHCl-based GPE	/	5.77	/	9
IL@MOF	0.33	7.5	800 h	10

Table S5. Comparison of cycling performance of different solid-state Al-graphite batteries.

Electrolyte	Maximum number of cycles	Maximum specific capacity (mA h g^{-1})	Ref.
**F-SSAF**	**10000**	**121**	**This work**
PA EMIC-GPE	4200	100	4
EMIC-based GPE	100	120	5
PA-Et_3NHCl-based GPE	2000	94.6	6
EA-EMIC-based GPE	500	90	8
Et_3NHCl-based GPE	800	90	9
IL@MOF	2000	75	10

Video S1. The F-SSAF-based pouch cells under harsh mechanical abuse.

Video S2. The F-SSAF electrolyte (left, white) and IL@GF/D (right) under a high-temperature flame (approximately 1000 ℃).

Supplementary References

(1) Frisch, M. J. et al. Gaussian 16 Rev. C.01. (2016).

(2) P. J. Stephens, F. J. Devlin, C. F. Chabalowski, M. J. Frisch, Ab initio calculation of vibrational absorption and circular dichroism spectra using density functional force fields. *J. Phys. Chem.* **1994**, *98*, 11623-11627.

(3) L. Goerigk, S. Grimme, Efficient and accurate double-hybrid-meta-GGA density functionals Evaluation with the extended GMTKN30 database for general main group thermochemistry, kinetics, and noncovalent interactions. *J. Chem. Theory Comput.* **2011**, *7*, 291-309.

(4) Liu, Z.; Du, H.; Cui, Y.; Du, L.; Zhao, Z.; Wang, X.; Lv, Z.; Sun, M.; Liu, Z.; Li, K.; A reliable gel polymer electrolyte enables stable cycling of rechargeable aluminum batteries in a wide-temperature range. *J. Power Sources* **2021**, *497*.

(5) Yu, Z.; Jiao, S.; Li, S.; Chen, X.; Song, W.-L.; Teng, T.; Tu, J.; Chen, H.-S.; Zhang, G.; Fang, D.-N. Flexible Stable Solid-State Al-Ion Batteries. *Adv. Funct. Mater.* **2019**, *29* (1), 1806799.

(6) Liu, Z.; Wang, X.; Liu, Z.; Zhang, S.; Lv, Z.; Cui, Y.; Du, L.; Li, K.; Zhang, G.; Lin, M. C.; Du, H. Low-cost gel polymer electrolyte for high-performance aluminum-ion batteries. *ACS Appl. Mater. Interfaces* **2021**, *13*, 28164-28170.

(7) Sun, X.-G.; Fang, Y.; Jiang, X.; Yoshii, K.; Tsuda, T.; Dai, S. Polymer gel electrolytes for application in aluminum deposition and rechargeable aluminum ion batteries. *Chem. Commun.* **2016**, *52* (2), 292-295.

(8) Kim, I.; Jang, S.; Lee, K. H.; Tak, Y.; Lee, G. In situ polymerized solid electrolytes for superior safety and stability of flexible solid-state Al-ion batteries. *Energy Storage Mater.* **2021**, *40*, 229-

238.

(9) Yu, Z.; Jiao, S.; Tu, J.; Song, W.-L.; Lei, H.; Jiao, H.; Chen, H.; Fang, D. Gel electrolytes with a wide potential window for high-rate Al-ion batteries. *J. Mater. Chem. A* **2019**, *7* (35), 20348-20356.

(10) Huang, Z.; Song, W. L.; Liu, Y.; Wang, W.; Wang, M.; Ge, J.; Jiao, H.; Jiao, S. Stable quasi-solid-state aluminum batteries. *Adv. Mater.* **2022**, *34*, e2104557.
