# Supplementary material for: A Recyclable Inert Inorganic Framework Assisted Solid-State Electrolyte for Long-Life Aluminum Ion Batteries
Source: ACS Cent Sci. 2024 Dec 19;11(2):239–47. doi: 10.1021/acscentsci.4c01615 (PMC11869131; doi:10.1021/acscentsci.4c01615)
Supplement: Supplementary file 4 — oc4c01615_si_004.pdf [file oc4c01615_si_004.pdf]

oc-2024-01615r.R1

Name: Peer Review Information for "A recyclable inert inorganic framework assisted solid-state electrolyte for long-life aluminum ion batteries"

First Round of Reviewer Comments

Reviewer: 1

Comments to the Author

This manuscript reported a novel recyclable inert inorganic framework for solid-state aluminum ion batteries (SSAIBs). Compared with the nonaqueous AIBs, the SSAF-based SSAIBs have the features of no leakage risk, high safety, inhibition of graphite cathode expansion but poor contact interface between electrodes and electrolyte. Therefore, the F-SSAF that inherits both FIL and SSAF is built, and high-performance SSAIBs are achieved. Overall, the manuscript shows very interesting results which prepares the field towards solid state Al batteries, and bears great significance for the field. However, I have some comments that should be clarified before acceptance. Detailed questions/comments are as following:

1. In this work, the authors used AlF_3 as inorganic framework and prepared a SSAF electrolyte by mixing AlF_3 with IL. Why did the authors choose AlF_3? Please provide the structure and the space size of AlF_3.

2. After adding the FEC additive, will it react with EMIC-$AlCl_3$? Please provide additional experimental data as evidence.

3. In Figure S4 g-h, the cycling processes of Al|3 vol% FEC@EMIC-$AlCl_3$|Al become unstable. The authors are suggested to provide further discussion on this point.

4. The authors stated that AlF_3 can be recycled in the electrolyte, which is interesting and economic. In Figure S22, the recycling yield of AlF_3 is reported to be over 80%. As an inert inorganic framework, its recycling yield may be more than this value. Please provide some explanations.

5. CV data are lacking in this work. Does the CV curve of SSAF differ from EMIC-$AlCl_3$? Additionally, would the introduction of the FEC additive also affect the CV behavior of the battery?

6. A graphite cathode with F-SSAF electrolyte exhibits a long cycle life of 10000 cycles with high capacity retention and Coulombic efficiency. Why did the capacity first increase and then decrease during the long cycling? And, what about the rate performance of Al|F-SSAF|graphite at high current densities (1 A g^{-1}, 2 A g^{-1}, 5 A g^{-1} etc.)?

Reviewer: 2

Comments to the Author

In this work, the authors described a solid-state electrolyte with AlF_3 inert inorganic framework as a solid diluent for solid-state aluminum-ion batteries. Furthermore, the interface additive was introduced into the EMIC-$AlCl_3$, achieving a stable cycling of Al||Al symmetric batteries for up to 4000 hours. I recommend this work to be published after minor revisions, and some other comments are given as below.

1 Full names of all the abbreviations should be given when they first appeared. Please check the whole paper.

2. The current work still needs more detailed analysis of the F-rich SEI and CEI at anode and cathode. Please provide the EIS data when the cell was charged to 2.4 V and discharged to 0.1 V (or close to 0.1 V) after different cycles and the explanations.

3 In lithium-ion batteries, only Li^+ diffusion occurs in the inorganic solid electrolyte, and theoretically, the ion transference number (t_+) is equal to 1. In Figure 1f, the ion transference number of the SSAF electrolyte is 0.5 and EMIC-$AlCl_3$ is 0.14. Some explanations are needed.

4 Could the authors identify other inorganic inert frameworks that serve as electrolyte materials? What are the common characteristics and distinctions among these materials?

4 It appears that XR-CT is a method designed for non-destructive analysis of solid electrolyte structure. Could the authors provide a description of the corresponding experimental details and the technical challenges?

5 Can the authors elaborate on the potential future directions for the development of aluminum-ion batteries in the introduction part?

Author's Response to Peer Review Comments:

To Reviewer 1:

Comments: This manuscript reported a novel recyclable inert inorganic framework for solid-state aluminum ion batteries (SSAIBs). Compared with the nonaqueous AIBs, the SSAF-based SSAIBs have the features of no leakage risk, high safety, inhibition of graphite cathode expansion but poor contact interface between electrodes and electrolyte. Therefore, the F-SSAF that inherits both FIL and SSAF is built, and high-performance SSAIBs are achieved. Overall, the manuscript shows very interesting results which prepares the field towards solid state Al batteries, and bears great significance for the field. However, I have some comments that should be clarified before acceptance. Detailed questions/comments are as following:

Response: We are thankful to you for the kind recommendation and careful review. According to your helpful suggestions, we have carefully revised the manuscript and made a point-by-point response as shown below. All changes have been highlighted in red in the revised manuscript and supplementary information.

Comment 1: In this work, the authors used AlF_3 as inorganic framework and prepared a SSAF electrolyte by mixing AlF_3 with IL. Why did the authors choose AlF_3? Please provide the structure and the space size of AlF_3.

Response 1: Thanks for your valuable comment. AlF_3 is an ideal framework material for stable electrolytes due to its outstanding thermal and chemical stability. These properties ensure that it remains stable in demanding electrolyte environments. Additionally, the structure of AlF_3 provides a large surface area and porosity, which can effectively accommodate EMIC-$AlCl_3$. Meanwhile, the dissociation of $Al_2Cl_7^-$ ($AlCl_3$-$AlCl_4^-$) into $AlCl_4^-$ is promoted by AlF_3, which can facilitate the migration rate of $AlCl_4^-$ active ions and simultaneously mitigate the corrosion of Al anode. When compared to other frameworks, such as gel electrolytes and metal-organic frameworks (MOFs), AlF_3 offers several key advantages. Its simple preparation process and low cost make it a more practical choice, especially for large-scale applications. And, we provide the structure and the space size of AlF_3 as shown in **Supplementary Figures S13 and S14**. AlF_3 crystallizes in a rhombohedral crystal structure at room temperature, with a trigonal space group R-3c. The framework is composed of corner-sharing AlF_6 octahedra, forming a three-dimensional network. This network not only contributes to its structural rigidity but also provides sufficient porosity to enable excellent

compatibility with ILs, further improving ion transport properties. We have added the description in the revised manuscript. Please see the following.

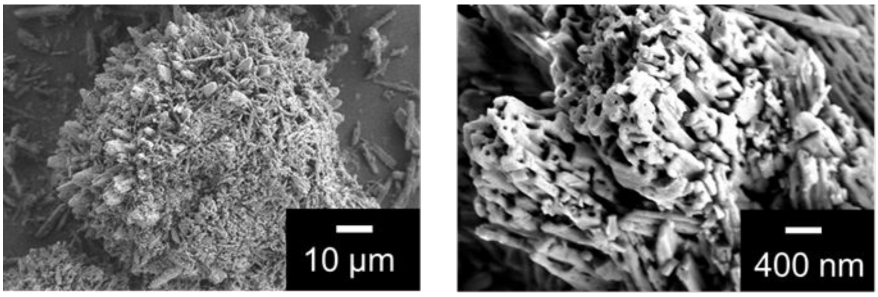

Supplementary Figure S13. The SEM images of AlF_3.

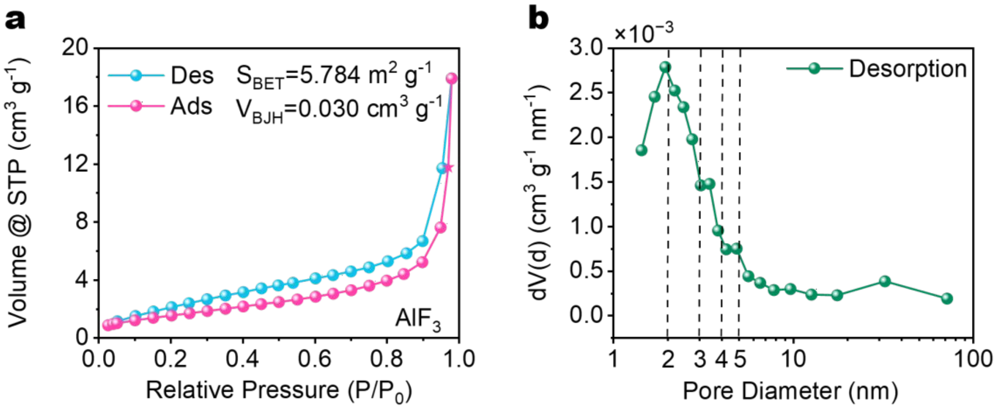

Supplementary Figure S14. The BET tests of AlF_3.

"AlF_3 is an ideal framework material for stable electrolytes due to its outstanding thermal and chemical stability. These properties ensure that it remains stable in demanding electrolyte environments. The scanning electron microscopy (SEM) and Brunauer–Emmett–Teller (BET) tests display porous AlF_3 microtubes, which possesses sufficient adsorption capacity for EMIC-$AlCl_3$ (Figures S13 and S14). The framework is composed of corner-sharing AlF_6 octahedra, forming a three-dimensional network. This network not only contributes to its structural rigidity but also provides sufficient porosity to enable excellent compatibility with EMIC-$AlCl_3$, further improving ion transport properties." (Please see Page 9, red-labeled part)

"One the one hand, the AlF_3 can promote the dissociation of $Al_2Cl_7^-$ ($AlCl_3$-$AlCl_4^-$) into $AlCl_4^-$, which can facilitate the migration rate of $AlCl_4^-$ active ions and enhance the ion transference number of $AlCl_4^-$. At the same time, the decrease of $Al_2Cl_7^-$ can mitigate the corrosion of Al anode." (Please see

Page 13, red-labeled part)

Comment 2. After adding the FEC additive, will it react with EMIC-$AlCl_3$? Please provide additional experimental data as evidence.

Response 2: Thanks for your valuable comment. We have performed more detailed spectral characterization to further clarify the difference between EMIC-$AlCl_3$ and FEC@EMIC-$AlCl_3$. Both FT-IR spectra and NMR results indicate that a chemical reaction took place between FEC and EMIC-$AlCl_3$ (**Supplementary Figure S5**). We have added the description in the revised manuscript. Please see the following.

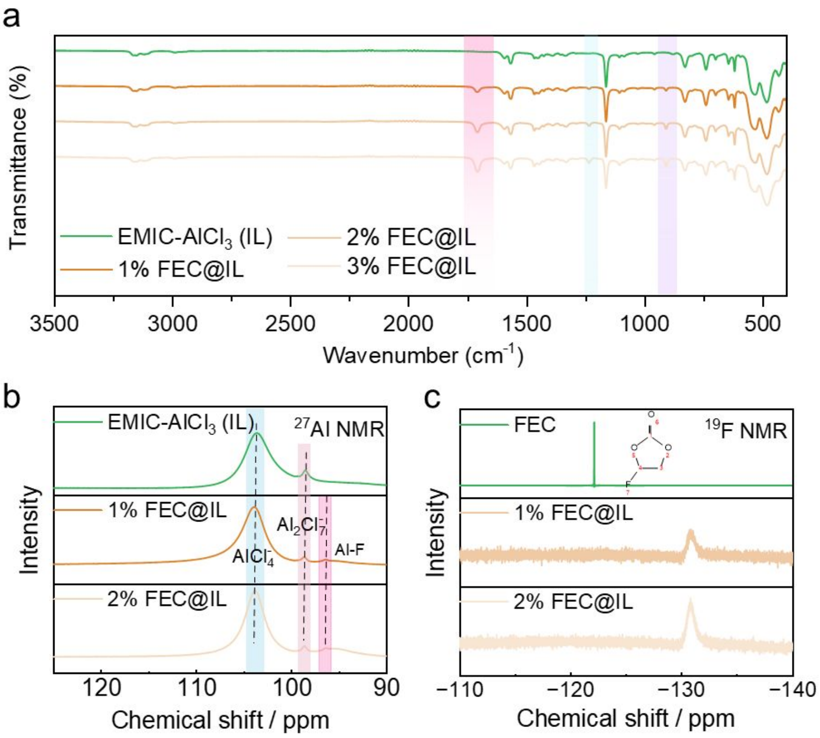

Supplementary Figure S5. (a) FT–IR spectra of EMIC-$AlCl_3$ (IL), 1% FEC@IL, 2% FEC@IL and 3% FEC@IL; (b) ^{27}Al NMR spectra of EMIC-$AlCl_3$ (IL), 1% FEC@IL and 2% FEC@IL; (c)^{19}F NMR of FEC, 1% FEC@IL and 2% FEC@IL.

"After adding the FEC additive in EMIC-$AlCl_3$, Fourier transform infrared (FT-IR) spectra show that C=O (1708 cm^{-1}), C-O-C (1235 cm^{-1}) and C-F (915 cm^{-1}) peaks are enhanced with increased FEC concentration. And ^{27}Al and ^{19}F NMR spectra also show that a new single Al-F signal, suggesting a polymerization reaction between FEC and EMIC-$AlCl_3$ (Figure S5). This is basically consistent with the reported literatures[24-25]." (Please see Page 6, red-labeled part).

(24) S. Sarkar, B. Chen, C. Zhou, S. N. Shirazi, F. Langer, J. Schwenzel, V. Thangadurai, Synergistic Approach toward Developing Highly Compatible Garnet-Liquid Electrolyte Interphase in Hybrid Solid-State Lithium-Metal Batteries. *Adv. Energy Mater.* **2023**, *13*, 2203897.

(25) Y. Zhang, Y. Wu, H. Li, J. Chen, D. Lei, C. Wang. A dual-function liquid electrolyte additive for high-energy non-aqueous lithium metal batteries. *Nat. Commun.* **2022**, *13* (1), 1297.

Comment 3. In Figure S4 g-h, the cycling processes of Al|3 vol% FEC@EMIC-$AlCl_3$|Al become unstable. The authors are suggested to provide further discussion on this point.

Response 3: Thanks for your valuable comment. Excessive addition of FEC leads to the consumption of $AlCl_3$ in the EMIC-$AlCl_3$, resulting in a decrease in the concentration of $Al_2Cl_7^-$, which affects the reversible deposition of aluminum (as described in reaction equation (1)). Therefore, the molar ratio of $AlCl_3$ and EMIC is set to be 1.5:1 to ensure that, even after the introduction of the FEC additive, the ratio remains above 1.3:1. Some explanations have been added in the revised manuscript. Please see the following.

$$4Al_2Cl_7^- + 3e^- \leftrightarrow Al + 7AlCl_4^- \qquad (1)$$

"Therefore, the excessive incorporation of FEC (3 vol%) into the EMIC-$AlCl_3$ electrolyte leads to a pronounced consumption of $AlCl_3$. This reduces the concentration of anions, which in turn adversely affects the reversibility of aluminum deposition." (Please see Page 6, red-labeled part).

Comment 4. The authors stated that AlF_3 can be recycled in the electrolyte, which is interesting and economic. In Figure S22, the recycling yield of AlF_3 is reported to be over 80%. As an inert inorganic framework, its recycling yield may be more than this value. Please provide some explanations.

Response 4: Thanks for your nice comment. The recycling process of AlF_3 mainly includes several steps such as filtration, washing, and drying. During these steps, although we have endeavored to optimize the experimental details and employ efficient equipment, some minor mechanical losses are inevitable, including losses during filtration and transfer as well as the loss of AlF_3 in the washing process. Nevertheless, our experimental data indicate that the influence of these mechanical losses is limited, and the overall recovery rate can still be stably maintained at over 80%. These losses are

attributable to small-scale laboratory experiments, where inefficiencies are more pronounced. In large-scale industrial production, improved process efficiency is expected to yield higher recovery rates. Further optimization of these steps, particularly through the improvement of equipment and strict control of the process, is anticipated to enhance the recovery rate. Some explanations have been added in the revised manuscript. Please see the following.

"It is worth noting that these losses are primarily associated with small-scale laboratory experiments, where unavoidable inefficiencies are more pronounced. In large-scale industrial production, the recovery rate is expected to be higher due to improved process efficiency." (Please see Page 12, red-labeled part).

Comment 5. CV data are lacking in this work. Does the CV curve of SSAF differ from EMIC-$AlCl_3$? Additionally, would the introduction of the FEC additive also affect the CV behavior of the battery?

Response 5: Thanks for your valuable comment. We have provided the CV data of the four electrolytes. The CV curves of SSAF and EMIC-$AlCl_3$ electrolytes are consistent, as both electrolytes utilize the same electrode material and the $AlCl_4^-$ intercalation processes are also similar. However, for the F-SSAF electrolyte, which incorporates a FIL interface into the SSAF, a prominent reduction peak is observed at 2.2 V. This peak corresponds to the high discharge plateau of the batteries. Therefore, this further confirms that the introduction of FEC additive into SSAF electrolyte surface enhances the stability of the electrolyte-electrode interface and F-rich SEI and CEI films are in-situ formed. The related explanations have been added in the revised manuscript. Please see the following.

"To analyze the electrochemical performance of the batteries, CV tests of EMIC-$AlCl_3$, FIL, SSAF and F-SSAF at 0.2, 0.4, 0.6 0.8, 1.0 mV s^{-1} were carried out (Figure S11). This further confirms that the introduction of FEC additive into SSAF electrolyte surface enhances ion transference ability and the stability of the electrolyte-electrode interface." (Please see Page 7, red-labeled part).

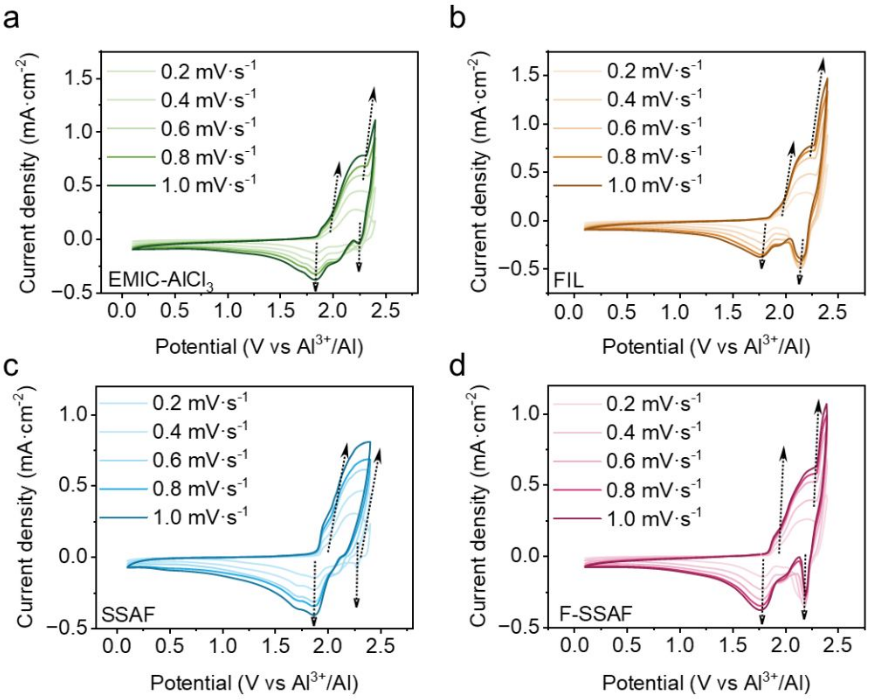

Supplementary Figure 11. CV curves of the EMIC-$AlCl_3$, FIL, SSAF and F-SSAF at 0.2, 0.4, 0.6 0.8, 1.0 mV s^{-1}.

Comment 6. A graphite cathode with F-SSAF electrolyte exhibits a long cycle life of 10000 cycles with high capacity retention and Coulombic efficiency. Why did the capacity first increase and then decrease during the long cycling? And, what about the rate performance of Al|F-SSAF|graphite at high current densities (1 A g^{-1}, 2 A g^{-1}, 5 A g^{-1} etc.)?

Response 6: Thanks for your valuable comment. We have observed a certain increase in capacity during the initial cycles, which can be attributed to the activation effect of the graphite electrode during cycling. This includes the gradual stabilization of the F-rich SEI and CEI films and the optimization of interfacial contact between electrolyte and electrode. The rate performances of Al|F-SSAF|graphite at high current densities have been added in the revised manuscript. Please see the following.

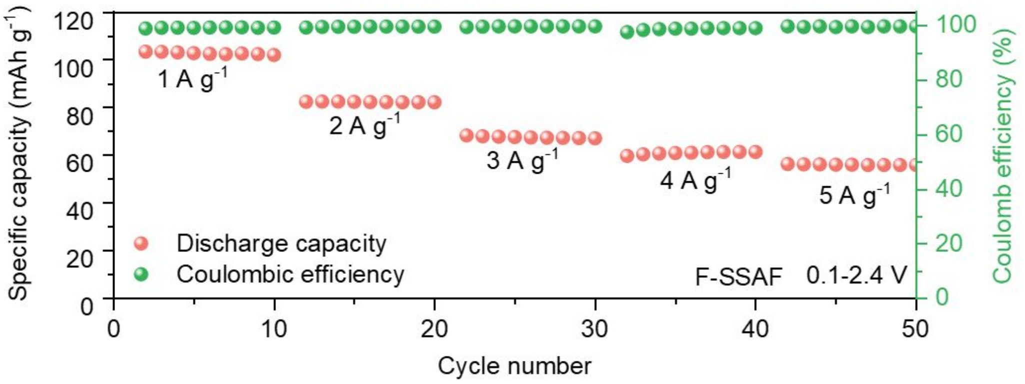

Supplementary Figure S12. Rate performance of F-SSAF with graphite cathode at various current densities from 1 to 5 A g^{-1}.

"Meanwhile, the Al||Graphite battery with F-SSAF electrolyte also delivers an excellent rate performance, even at a high current density of 5 A g^{-1} (Figure S12)." (Please see Page 7-8, red-labeled part).

To Reviewer 2:

Comments:

In this work, the authors described a solid-state electrolyte with AlF_3 inert inorganic framework as a solid diluent for solid-state aluminum-ion batteries. Furthermore, the interface additive was introduced into the EMIC-$AlCl_3$, achieving a stable cycling of Al||Al symmetric batteries for up to 4000 hours. I recommend this work to be published after minor revisions, and some other comments are given as below.

Response: We are thankful to you for the kind recommendation and careful review. All main revisions are marked with red font in the revised manuscript and supplementary information.

Comment 1: Full names of all the abbreviations should be given when they first appeared. Please check the whole paper.

Response 1: Thanks for your nice suggestion. We have carefully reviewed the entire paper and ensured that all abbreviations are now introduced with their full names upon first mention.

Comment 2: The current work still needs more detailed analysis of the F-rich SEI and CEI at anode and cathode. Please provide the EIS data when the cell was charged to 2.4 V and discharged to 0.1 V (or close to 0.1 V) after different cycles and the explanations.

Response 2: Thanks for your valuable comment. We have provided EIS data (**Supplementary Fig 22**) when the cell is charged to 2.4 V and discharged to 0.1 V after different cycles (1st cycle, 3rd cycle, 5th cycle), which also show a consistency and stable layer during cycling. Please see the following.

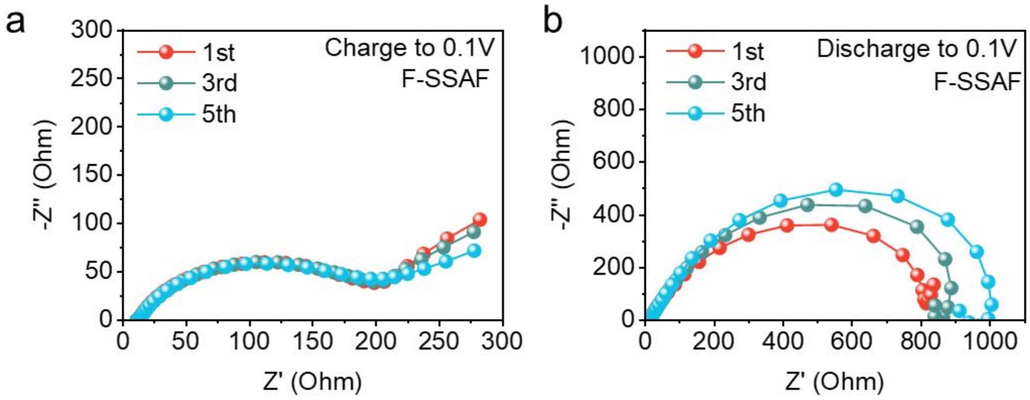

Supplementary Figure S22. (a) Nyquist plots of Al|F-SSAF|PG charged to 2.4V after different cycles. (b) Nyquist plots of Al|F-SSAF|PG discharged to 0.1V after different cycles.

"EIS data of different cycles are consistent, also indicating the formation of stable interfacial layers (Figure S22)." (Please see Page 10, red-labeled part).

Comment 3: In lithium-ion batteries, only Li^+ diffusion occurs in the inorganic solid electrolyte, and theoretically, the ion transference number (t^+) is equal to 1. In Figure 1f, the ion transference number of the SSAF electrolyte is 0.5 and EMIC-$AlCl_3$ is 0.14. Some explanations are needed.

Response 3: Thanks for your insightful observation regarding the ion transference numbers (t^+) presented in **Figure 1f**. In the EMIC-$AlCl_3$ electrolyte, anions ($AlCl_4^-$, $Al_2Cl_7^-$) exhibit a degree of mobility; however, their migration is significantly hindered by strong interactions with cations (EMI^+), resulting in limited dissociation due to the intense ionic interactions. In contrast, the SSAF electrolyte promotes the dissociation of $Al_2Cl_7^-$ ($AlCl_3$-$AlCl_4^-$) into $AlCl_4^-$, facilitated by the presence of AlF_3, which enhances the migration rate of $AlCl_4^-$ active ions. Raman spectra comparisons between EMIC-

$AlCl_3$ and FIL electrolytes reveal characteristic peaks corresponding to $AlCl_4^-$ (349 cm^{-1}), $Al_2Cl_7^-$ (310 cm^{-1}), and EMI^+ (597 cm^{-1}). However, in SSAF and F-SSAF electrolytes, the $Al_2Cl_7^-$ peak disappears (Figure 2f, g). Therefore, the ion transference number of the SSAF electrolyte is higher than EMIC-$AlCl_3$. For an all-solid-state electrolyte, only Li^+ diffusion occurs. However, the SSAF electrolyte is not an all-solid-state electrolyte, and thus exhibits ion transference number is lower than 1. Some explanations have been added in the revised manuscript. Please see the following.

"One the one hand, the AlF_3 can promote the dissociation of $Al_2Cl_7^-$ ($AlCl_3$-$AlCl_4^-$) into $AlCl_4^-$, which can facilitate the migration rate of $AlCl_4^-$ active ions and enhance the ion transference number of $AlCl_4^-$. At the same time, the decrease of $Al_2Cl_7^-$ can mitigate the corrosion of Al anode." (Please see Page 13, red-labeled part)

Comment 4: Could the authors identify other inorganic inert frameworks that serve as electrolyte materials? What are the common characteristics and distinctions among these materials?

Response 4: Thanks for your nice suggestion. We agree that exploring other inorganic inert frameworks as electrolyte materials. Below, we show several examples (LiF, KF, NaF, Al_2O_3) and discuss their common characteristics and distinctions:

An ideal framework must exhibit excellent chemical and electrochemical stability and proper surface area. The primary requirement is to ensure the reversibility of Al deposition/dissolution and the stability of the cyclic voltammetry (CV) curve in the Al|Electrolyte|Graphite batteries. For instance, while the LiF@EMIC-$AlCl_3$ electrolyte demonstrates reversible Al deposition/dissolution stability, its CV test stability is poor (**Figure R1(a, d)**). Similarly, NaF@EMIC-$AlCl_3$ and KF@EMIC-$AlCl_3$ exhibit poor Al deposition/dissolution stability with obvious side reactions, resulting in unstable CV curves (**Figure R1(b, c, e, f)**). In contrast, the Al_2O_3@EMIC-$AlCl_3$ framework offers diverse morphologies and interface tunability. As a solid framework, it demonstrates excellent Al deposition/dissolution stability and stable CV test performance (**Figure R1(g, h)**). Moreover, it delivers a high initial specific capacity at 50 mA g^{-1} (**Figure R1(i)**), but its long-term cycling stability is unsatisfactory. Looking ahead, our research is dedicated to identifying and developing novel high-stability solid electrolyte frameworks for aluminum-ion batteries (AIBs), which will serve as a critical factor in achieving enhanced long-term cycling stability.

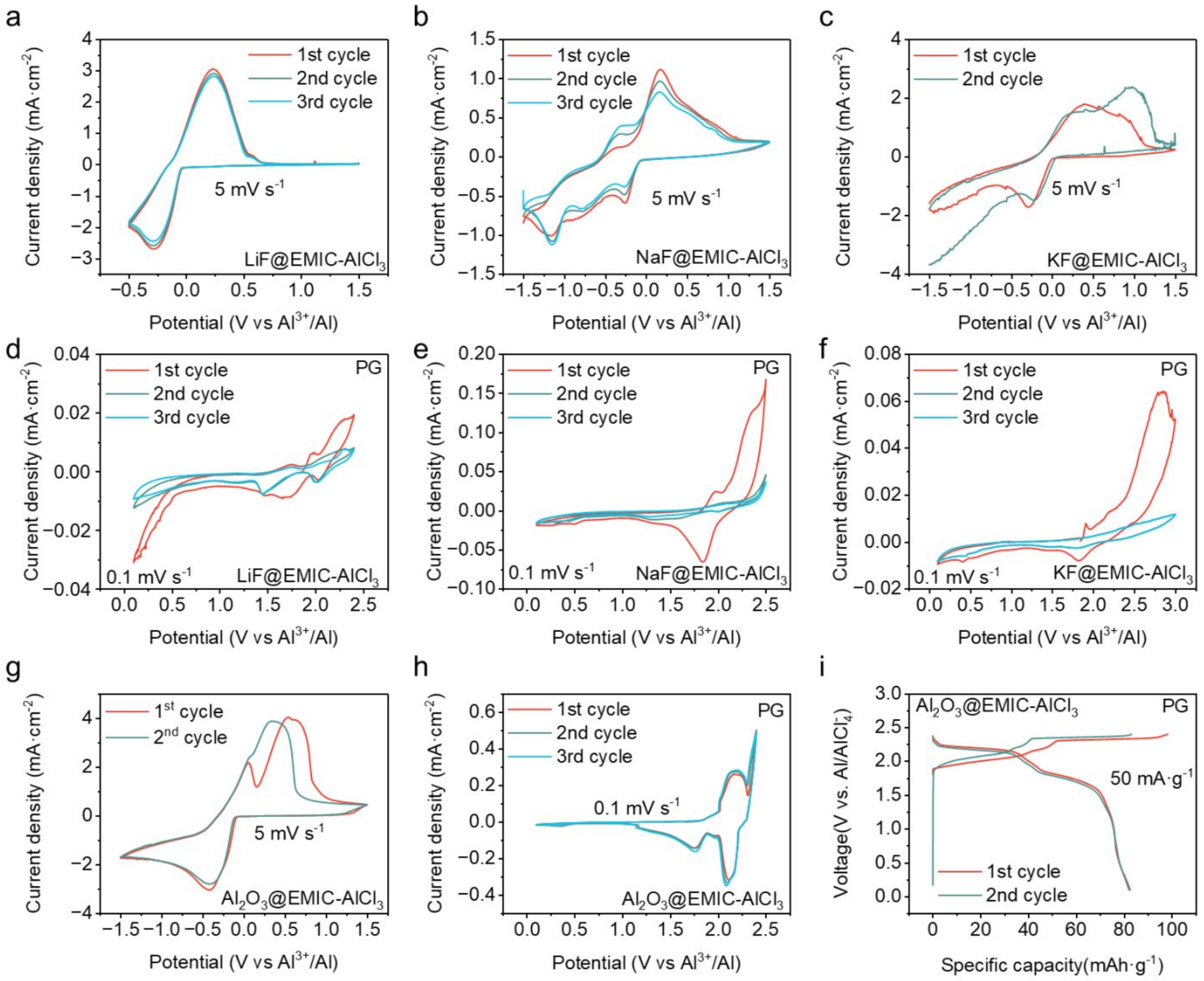

Figure R1. CV curves of the Al||Mo cells (a-c, g) and Al||PG cells (d-e, h) using LiF@EMIC-$AlCl_3$, NaF@EMIC-$AlCl_3$, KF@EMIC-$AlCl_3$ and Al_2O_3@EMIC-$AlCl_3$ electrolyte; (i) The charge/discharge voltage profiles of Al|Al_2O_3@EMIC-$AlCl_3$|PG cells of 50 mA g^{-1}.

Comment 5: It appears that XR-CT is a method designed for non-destructive analysis of solid electrolyte structure. Could the authors provide a description of the corresponding experimental details and the technical challenges?

Response 5: Thanks for your valuable comment. We have added a description of the corresponding experimental details and the technical challenges in the revised manuscript and revised supplement information. Please see the following.

"However, several technical challenges remain to be addressed, such as the limited imaging volume of high-resolution CT, the time-intensive multi-angle data acquisition unsuitable for rapid dynamic processes, and the substantial computational and expertise demands for accurate reconstruction and

segmentation." (Please see Page 8, red-labeled part).

"XR-CT experiments were performed using an Xradia 520 Versa system (Zeiss). To achieve sufficient resolution, a customized Swagelok cell with a small diameter was designed and fabricated. This cell ensured both gas impermeability and a clear density contrast between the electrolyte and the Swagelok (PTFE material). The density difference was essential for effectively distinguishing the solid-state electrolyte framework from the Swagelok components during image analysis. The CT system acquired X-ray projection data from multiple angles, which was then processed via computational reconstruction to generate cross-sectional and 3D images." (Please see Page 3-4, red-labeled part in the revised supplement information).

Comment 6: Can the authors elaborate on the potential future directions for the development of aluminum-ion batteries in the introduction part?

Response 6: Thanks for your nice comment. The potential future directions for the development of aluminum-ion batteries have been added in the introduction part of the revised manuscript. Please see the following.

"Future directions for the development of AIBs will focus on improving energy density, cycle life, and electrolyte stability, while the development of advanced electrodes, scalable production, and cost-effective solutions will be key to practical applications." (Please see Page 4, red-labeled part).

Hopefully, our Reply and revision have well cleared all the concerns upon reading the manuscript. Again, many thanks for help in improving our work.

Sincerely yours,

Shuqiang Jiao
Professor of Electrochemical Engineering
State Key Laboratory of Advanced Metallurgy
University of Science and Technology Beijing, Beijing 100083, China.
Email: sjiao@ustb.edu.cn
Tel&Fax: +86-10-62333617
Dec. 2, 2024
